# Brevetoxin Metabolites: Emerging Toxins in French Shellfish Determined by LC-MS/MS and ELISA

**DOI:** 10.3390/md24020067

**Published:** 2026-02-03

**Authors:** Zouher Amzil, Amélie Derrien, Korian Lhaute, Aouregan Terre Terrillon, Simon Tanniou

**Affiliations:** 1 Ifremer (French Research Institute for Exploitation of the Sea)/PHYTOX/METALG, F-44311 Nantes, France; korian.lhaute@ifremer.fr (K.L.); simon.tanniou@ifremer.fr (S.T.); 2 Ifremer (French Research Institute for Exploitation of the Sea)/COAST/LER-BO, F-29900 Concarneau, France; amelie.derrien@ifremer.fr (A.D.); aouregan.terre.terrillon@ifremer.fr (A.T.T.)

**Keywords:** brevetoxins, LC-MS/MS, ELISA Kit, mollusks

## Abstract

In France, as part of the monitoring program for the emergence of marine toxins in shellfish (EMERGTOX), brevetoxins (BTX-2, BTX-3) were first detected in shellfish from Corsica (Mediterranean Sea) in 2018. The complex metabolic transformation of brevetoxins in shellfish, coupled with the limited availability of analytical standards for most metabolites, complicates the accurate evaluation of contamination levels. To address this challenge, two complementary analytical approaches were implemented to quantify brevetoxin metabolites in shellfish samples collected from 2018 to 2023: (i) a targeted LC-MS/MS method specially developed for brevetoxins; and (ii) an ELISA capable of detecting metabolites for which no reference standards are available. Of the 11 brevetoxin metabolites targeted, 4 were quantified by LC-MS/MS: BTX-2, BTX-3, BTX-B5, and S-deoxy-BTX-B2 (including its isomers). The ELISA consistently detected brevetoxins in all Corsican samples previously confirmed positive by LC-MS/MS, with concentrations systematically exceeding those measured by LC-MS/MS. This overestimation may result from antibody cross-reactivity and from the presence of unidentified brevetoxin metabolites not detected by LC-MS/MS. Regardless of the analytical method used, the highest concentration detected exceeded the current French guideline value for brevetoxins in shellfish. To ensure consumer protection, a two-step monitoring strategy is proposed: initial screening via ELISA to estimate brevetoxin contamination, followed by confirmatory LC-MS/MS analysis to identify and quantify the specific metabolites.

## 1. Introduction

**Figure 1:**
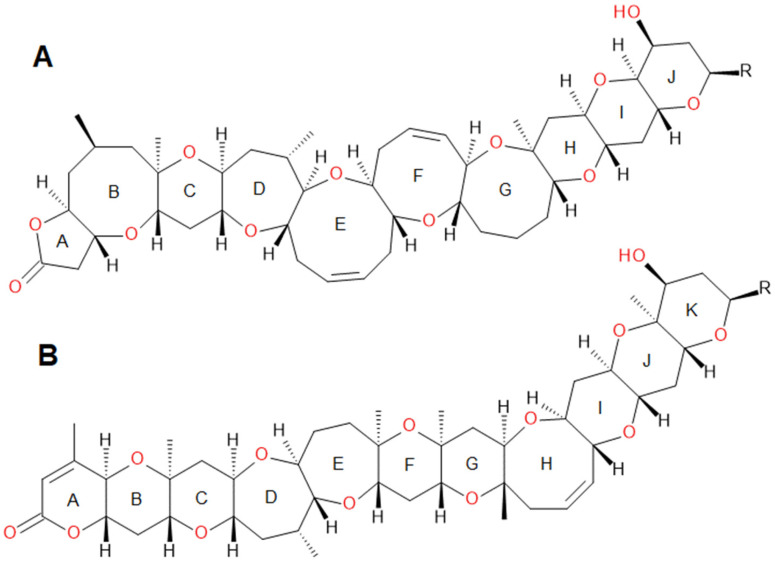
Structures of brevetoxin A (**A**) and B (**B**) backbones [7].

Brevetoxins (BTXs), produced by the dinoflagellate *Karenia brevis* (formerly known as *Gymnodinium breve* Davis and *Ptychodiscus brevis* Steidinger), are lipid-soluble and heat-stable cyclic polyether compounds [1]. Two distinct structural backbones have been characterized: type A and type B brevetoxins, containing 10 or 11 rings, respectively (Figure 1) [2,3,4,5]. Both structures feature a lactone moiety, which is putatively essential for their biological activity [6]. The species *K. brevis* produces approximately 19 brevetoxin metabolites, including 7 type A metabolites. Several BTXs known to be produced by *K. brevis* have also been identified in shellfish, and they can undergo metabolic transformation in shellfish. A wide diversity of brevetoxin analogs has been identified to date [5].

*K. brevis* can proliferate extensively, leading to significant public-health and environmental impacts. Brevetoxins can (i) induce neurotoxic shellfish poisoning (NSP) in humans following the consumption of contaminated shellfish [8]; (ii) cause respiratory and cutaneous irritation among coastal water users [9,10,11,12]; and (iii) induce mass mortality in marine fauna due to their ichthyotoxic effects, thereby impacting wild populations and aquaculture activities, including fish and shellfish farming [13,14,15].

The species *K. brevis* has been recorded in Florida, the Gulf of Mexico, and the West Indies [16]. Although the responsible species was not definitively identified at the time, the event was linked to a *Karenia*-type gymnodinioid species capable of producing brevetoxins [16]. In these regions, the closure of shellfish production areas is determined by *Karenia* spp. cell counts or brevetoxin analysis in shellfish tissues. A maximum regulatory limit of 20 mouse units per 100 g of tissue, equivalent to 0.8 mg BTX-2 equivalents/kg of total shellfish flesh, is applied using the mouse bioassay (MBA) or 0.8 mg BTX-2 equivalents/kg of total shellfish flesh using the enzyme-linked immunosorbent assay (ELISA), as described in the National Shellfish Sanitation Program [17]. The most severe NSP outbreaks were reported in New Zealand between 1992 and 1993, resulting in over 180 human poisoning cases linked to the consumption of contaminated shellfish (mussels, cockles, and oysters) [18,19]. Clinical symptoms occur within 24 h after ingestion and may include gastrointestinal distress, paresthesia, ataxia, bradycardia, dizziness, loss of coordination and, in severe cases, coma [8,20]. To date, no human fatalities due to inhalation exposure to BTXs have been reported.

**Figure 2:**
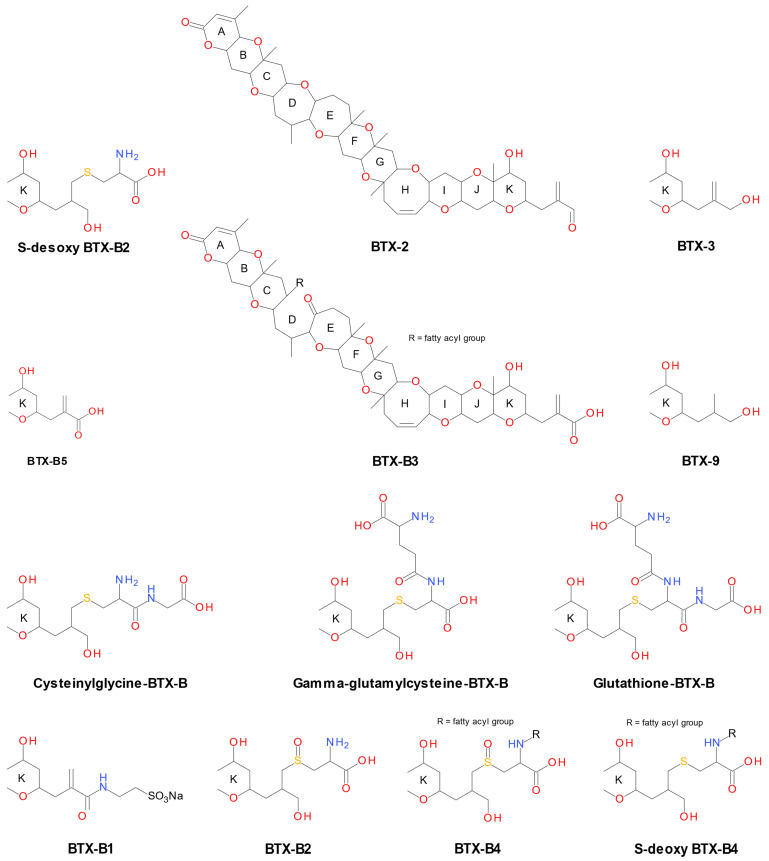
Main BTX-2 metabolites (lipophilic, polar) identified in shellfish [22].

Brevetoxins of algal origin can undergo metabolic transformation in shellfish. Following the identification of brevetoxin metabolites in the urine of individuals poisoned by BTX-3-contaminated shellfish (whelks, clams) [21], research efforts to characterize these compounds have intensified. Subsequent studies on both naturally and experimentally contaminated shellfish revealed that these metabolites result from oxidation, reduction, hydrolysis, or conjugation with endogenous molecules such as taurine, cysteine, cysteine sulfoxide, amino acids, and fatty acids [20,22]. These metabolites have not been detected in *K. brevis* cells, whether in laboratory cultures or during natural blooms. Figure 2 shows the primary brevetoxin metabolites formed in shellfish flesh, derived from the algal toxin BTX-2.

The metabolites derived from brevetoxin biotransformation in shellfish can exhibit increased polarity or lipophilicity relative to their precursor compounds. Conjugation with amino acids or peptides yields more polar metabolites, whereas fatty acid conjugation produces more lipophilic derivatives [5,23]. The presence of polar brevetoxin metabolites has been documented in oysters exposed to *K. brevis* blooms in situ, as well as in oysters experimentally contaminated with *K. brevis*. Accumulated BTX-2 was rapidly converted into a mixture of polar metabolites, which were subsequently eliminated at a slower rate. In contrast, approximately 90% of accumulated BTX-3 was eliminated within two weeks of exposure, with no evidence of biotransformation [24,25].

The scientific literature indicates that type B brevetoxin metabolites (e.g., BTX-2) are predominant in shellfish compared to type A metabolites (e.g., BTX-1, BTX-7). Specifically, distinct metabolites have been characterized in cockles (*Austrovenus stutchburyi*), including BTX-B1 (taurine–BTX-2 conjugate), and in green-shell mussels (*Perna canaliculus*), such as BTX-B2 (sulfoxidized cysteine–BTX-2), BTX-B3 (fatty acid conjugate of the open form of BTX-2), and BTX-B4 (N-palmitoyl–BTX-B2) [1,22,23,26,27,28,29,30,31].

In France, brevetoxins represent an emerging risk, and more broadly across Europe, they remain unregulated. Through the French monitoring program for emerging marine toxins in shellfish (EMERGTOX), BTX-2 and BTX-3 were first detected in autumn 2018 in Mediterranean mussels (*Mytilus*) from Corsica [32]. In response to this emerging threat, a dedicated working group was established in 2020 by the French Agency for Food, Environmental and Occupational Health & Safety (ANSES) to assess and prevent health risks associated with the consumption of BTX-contaminated shellfish [33,34]. An indicative safety threshold of 180 µg BTX-3 equation/kg of shellfish flesh was proposed, based on a default consumption portion of 400 g of shellfish flesh. Additionally, a priority list of brevetoxin analogs was established for targeted screening, based on available reference materials and toxicity data. This list includes BTX-1, BTX-2, BTX-3, taurine–BTX-B (BTX-B1), cysteine–BTX-B sulfoxide (BTX-B2), cysteine–BTX-B (S-deoxy-BTX-B2), oxidized D-ring-opened tetradecanoyl–BTX-2 (BTX-B3a), D-ring-opened hexadecanoyl–BTX-2 (BTX-B3b), N-tetradecanoyl–cysteine–BTX-B sulfoxide (N-myristoyl-BTX-B/BTX-B4a), N-hexadecanoyl–cysteine–BTX-B sulfoxide (N-palmitoyl–BTX-B2/BTX-B4b), and BTX-B5 (oxidized BTX-2) [34].

The concentrations of brevetoxins (BTXs) detected by LC-MS/MS in Corsican mussels between 2018 and 2023 [35] were consistently below the guidance value proposed by ANSES [33]. Nevertheless, the total BTX content may be significantly underestimated, as only two brevetoxin metabolites (BTX-2, BTX-3) were targeted among the potentially toxic analogs, using the lipophilic multi-toxin LC–MS/MS method applied in the EMERGTOX network (covering okadaic acid, dinophysistoxins, pectenotoxins, yessotoxins, azaspiracids, brevetoxins “BTX-2 & 3”, spirolides, pinnatoxins, gymnodimines, palytoxin and ovatoxins, domoic acid, and microcystins). To address this limitation, two complementary analytical approaches were implemented to quantify brevetoxin metabolites: (i) an ELISA-based screening assay (Abraxis) was used to detect type B brevetoxin metabolites in shellfish, including those not quantifiable by LC-MS/MS due to the absence of commercial standards and (ii) a targeted LC-MS/MS method, specifically developed for brevetoxins, was applied to quantify metabolites for which analytical standards are available.

A retrospective screening of all archived EMERGTOX samples from Corsica collected since 2018 was conducted using both complementary analytical approaches. In addition, samples from other mollusk species—including mussels, oysters, and clams—collected at various French coastal sites outside Corsica were also analyzed.

## 2. Results

### 2.1. Development, Optimization, and Implementation of a Brevetoxin-Specific LC-MS/MS Analytical Method

An extensive optimization process was conducted to define mass spectrometry parameters and chromatographic conditions for the targeted brevetoxin metabolites. This included the acquisition of reference fragmentation spectra, particularly for metabolites lacking commercially available standards but with known molecular masses [5]. Among the 11 metabolites targeted, only five reference standards were commercially available (BTX-1, BTX-2, BTX-3, BTX-B5, and S-deoxy-BTX-B2).

#### 2.1.1. Optimization of Mass Spectrometer Parameters

- Effect of infusion solution composition

BTX-1 was infused into thirteen distinct solvent compositions to evaluate their impact on ionization efficiency. For each experimental condition, the declustering potential (DP) was systematically optimized for the protonated [M + H]^+^, sodiated [M + Na]^+^, and ammoniated [M + NH_4_]^+^ ions (Table 1). The relative intensities of these precursor ions were compared to assess ionization efficiency and detection sensitivity under optimal instrumental settings.

**Figure 3:**
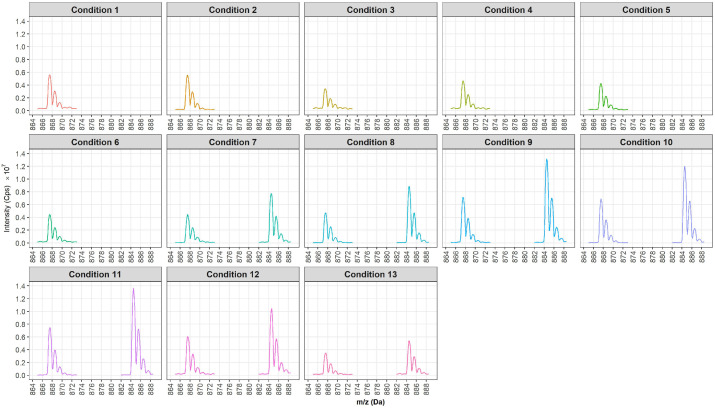
Mass spectrum (Q1) in positive ionization mode of BTX-1 in solution, using various infusion eluents.

Under all tested conditions, the [M + Na]^+^ ion exhibited low signal intensity and resistance to fragmentation, leading to its exclusion from further analyses. Figure 3 illustrates the mass spectral intensities of the [M + H]^+^ (m/z 867.5) and [M + NH_4_]^+^ (m/z 884.5) ions of BTX-1, obtained after DP optimization.

- Absence of Ammonium Formate or Ammonium Hydroxide (Acidic pH; Conditions 1–6)

In the absence of ammonium formate or ammonium hydroxide, only the [M + H]^+^ ion was detected. Acetonitrile (ACN, Conditions 1–3) yielded slightly higher signal intensities than methanol (MeOH, Conditions 4–6), particularly in Conditions 1 and 2. However, varying the formic acid concentration (0.1% to 0.3%) had no significant impact on ion sensitivity, regardless of the solvent used.

- Presence of Ammonium Formate (Acidic pH; Conditions 7–11)

The addition of ammonium formate promoted the preferential formation of [M + NH_4_]^+^ ions, which exhibited higher signal intensities than the [M + H]^+^ ions. Among the solvents tested, methanol-based solvents (Conditions 9–10) provided higher signal intensities for both ion types compared to acetonitrile-based solvents (conditions 7–8). Although isopropanol—known for its ability to enhance selectivity and resolution for certain analytes—was added to methanol (Condition 11), no significant improvement in sensitivity was observed. Furthermore, increasing the ammonium formate concentration from 2 mM to 5 mM did not result in a notable increase in ion intensity.

- Presence of Ammonium Hydroxide (Basic pH; Conditions 12–13)

Under basic conditions, the [M + NH_4_]^+^ ions displayed higher intensities than the [M + H]^+^ ions, particularly in acetonitrile (Condition 12). However, overall signal intensities remained lower compared to those obtained under acidic conditions with ammonium formate (Conditions 7–11).

- Optimization of Declustering Potential (DP), Collision Energy (CE), and Cell Exit Potential (CXP)

**Figure 4:**
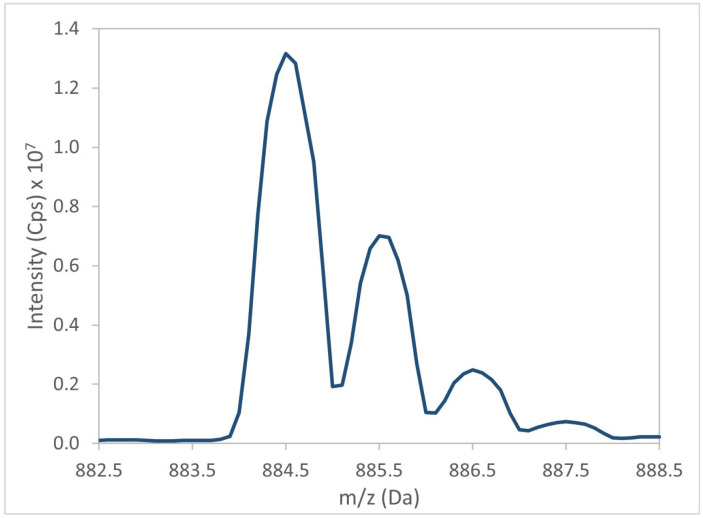
Mass spectrum (Q1) for BTX-1 ([M + NH_4_]^+^ ion).

**Figure 5:**
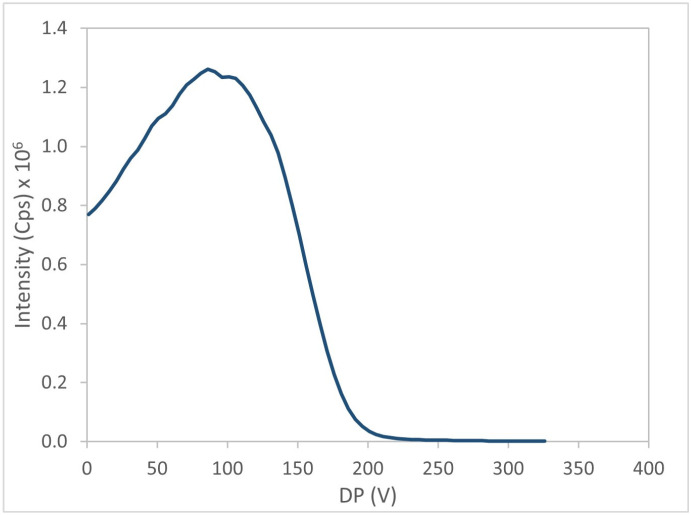
Evolution of signal intensity as a function of DP for BTX-1.

**Figure 6:**
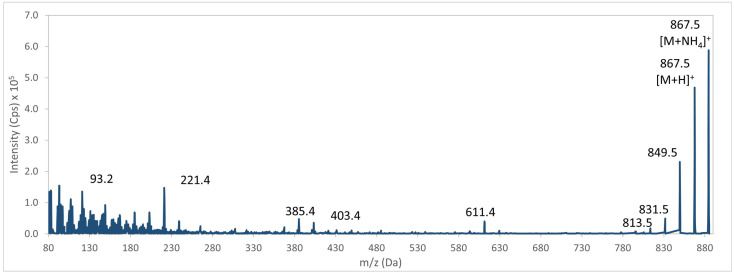
MS/MS fragmentation spectrum of BTX-1: Fragmentation of the precursor ion in Q2 and scan of the ions produced in Q3.

The mass spectrometer acquired a mass spectrum of the target precursor ion (e.g., BTX-1, Figure 4). The DP was then optimized to maximize signal intensity of precursor ion (Figure 5). Subsequently, the precursor ion was fragmented to generate an MS/MS spectrum of BTX-1 (Figure 6).

**Figure 7:**
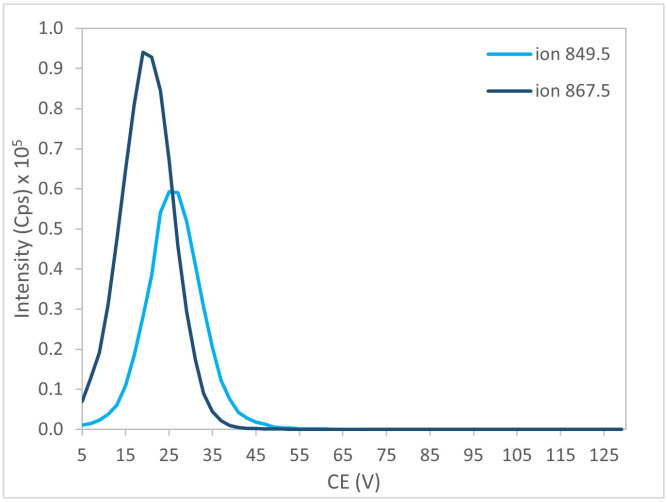
Evolution of signal intensity as a function of CE for the two selected daughter ions of BTX-1.

**Figure 8:**
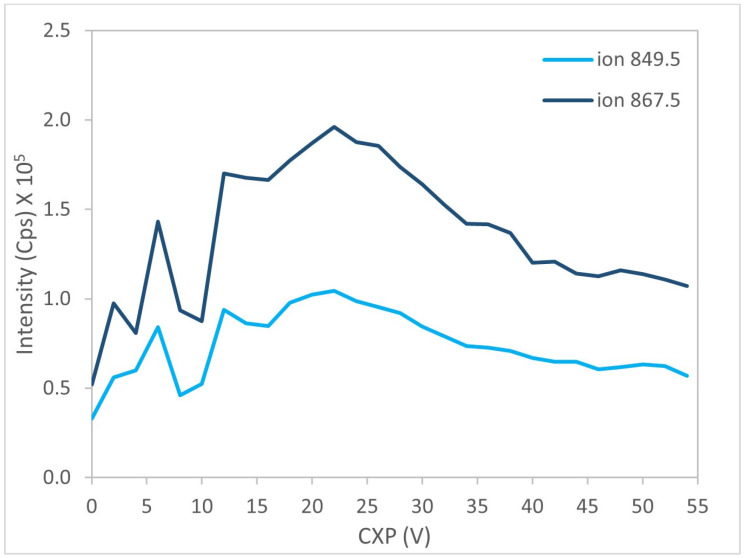
Evolution of signal intensity as a function of CXP for the two fragments ions of BTX-1.

Further optimization was conducted on the most abundant product ions by progressively adjusting the CE and CXP parameters. Figure 7 and Figure 8 illustrate the results obtained for the two selected ions of BTX-1.

BTX1 was included as an illustrative example, and the observations made for this compound were consistent with those for other BTX analogs. MS/MS fragmentation spectra for other BTXs with available reference materials (BTX-2, BTX-3, BTX-B5, and S-desoxy-BTX-B2) are provided as Supplementary Material (Figure S1). Although BTX-1 (type A) is described in detail as an example, comparable MS-dependent trends were observed for all type B BTXs analyzed, which are the relevant analogs detected in French shellfish.

Additionally, it was observed that the S-deoxy-BTX-B2 metabolite did not form an [M + NH_4_]^+^ adduct. Consequently, optimization was therefore performed using the [M + H]^+^ ion, and the resulting parameters were extended to other structurally similar metabolites (e.g., BTX-B1, BTX-B4a, and BTX-B4b, all containing sulfur). However, should commercial standards for these toxins become available, the relevance of this ionization strategy would need to be reassessed. The optimal DP, CE, and CXP parameters for all toxins are listed in Table 2.

- Optimization of source parameters: flow injection analysis (FIA)

Unlike the optimized compound-dependent parameters, the source parameters must remain common to all targeted toxins to ensure consistent analytical performance. For flow injection analysis (FIA), a flow rate of 0.2 mL/min was set. The mobile phase consisted of an aqueous eluent A (H_2_O) and an organic eluent B (95% MeOH), each containing 0.2% formic acid and 2 mM ammonium formate (Condition 9, Table 2).

An optimization solution containing four toxin standards (BTX-1, BTX-2, BTX-3 and BTX-B5) was repeatedly injected to determine the optimal source settings. The S-deoxy-BTX-B2 standard, which was only received at the end of the method development phase, was not included in this phase.

During the injections, source parameters—including ion spray (IS) voltage, temperature (TEM), desolvation gas (GS2), nebulizing gas (GS1) and curtain gas (CUR)—were individually adjusted to determine their optimal values. Figure S2 illustrates the evolution of the signal area as a function of the optimized parameters for BTX-1 (884.5 > 867.5) and BTX-2 (912.5 > 895.5). The final optimized parameters are listed in Table 3.

#### 2.1.2. Optimization of Liquid Chromatography (LC) Parameters

- Optimization of eluent composition

The eluent conditions previously described (§ 2.1.1., Table 1) enabled the identification of an optimal mobile phase composition for the chromatographic elution. This mobile phase consists of an aqueous eluent A (H_2_O) and an organic eluent B (95% MeOH), each supplemented with 0.2% formic acid and 2 mM ammonium formate.

- Selection of the chromatographic column

A total of ten chromatographic columns with varying dimensions, particle sizes, pore sizes, carbon loadings, and grafting types were evaluated (Table S1). Figure S3 shows the analytical chromatograms of a brevetoxin standard mixture at a concentration of approximately 100 ng/mL.

Chromatographic columns with dimensions of 100 × 2.1 mm, 1.7 µm were excluded due to the excessive backpressure they generated. Although the Kinetex F5 phase provided partial resolution of BTX-B5 and BTX-3, the separation of these analytes remained challenging. Additionally, the Kinetex F5, Kinetex C18, Polar C18, and Kinetex EVO-C18 phases exhibited lower analytical performance, particularly in terms of signal-to-noise ratios, and were therefore discarded.

Among the evaluated columns, the Kinetex XB-C18 50 x 2.1 mm, 1.7 µm afforded slightly superior peak resolution compared to the same phase with dimensions of 100 × 2.1 mm, 2.6 µm, while maintaining comparable signal-to-noise ratios. Therefore, Kinetex XB-C18 (50 × 2.1 mm, 1.7 µm) was selected for the analysis of BTX metabolites.

- Optimization of eluent gradient, oven temperature, and flow rate

To maximize separation efficiency, chromatographic resolution, and signal-to-noise ratio, an extensive evaluation of nearly thirty experimental conditions was conducted. The optimal chromatographic conditions were determined as follows: the gradient elution profile was established with an initial linear increase from 30% to 70% B over 1 min, followed by a gradual rise to 95% B over 9 min. The gradient was then brought to 100% B in 0.1 min and maintained at this level for 1.9 min before returning to 30% B in 0.1 min, with a final 3.9 min equilibration period. The flow rate was set at 0.3 mL/min, while the column temperature was maintained at 30 °C. Finally, a sample injection volume of 5 µL was used for all analyses.

- Evaluation of linearity and determination of detection and quantification limits

The linearity of the detector response was evaluated using certified standard brevetoxin solutions at increasing concentrations (Table S2). Five independent replicate injections were performed on non-consecutive days to ensure robustness. Calibration curves were established by plotting peak areas against concentrations using the ordinary least squares (OLS) regression method. Linearity was confirmed through analysis of variance (ANOVA, Fisher’s F-test). All acceptance criteria were fulfilled for all brevetoxins, confirming a linear relationship between analyte concentration and instrumental response. Detailed results for each toxin are shown in Figure S2.

To estimate the limits of detection (LODs) and quantification (LOQs), a signal-to-noise (S/N) ratio-based approach was applied. According to this method, a signal is considered detectable (LOD) at S/N ≥ 3, and quantifiable (LOQ) at S/N ≥ 10. To ensure the reliability of LOQ values, the estimated concentrations were also verified to fall within ±20% of the nominal values. These analyses were performed using low-concentration standards. The resulting LOD and LOQ values are summarized in Table S3.

For brevetoxin metabolites lacking available reference materials (BTX-B3a, BTX-B3b, BTX-B1, BTXB2, BTX-B4a, BTX-B4b, and BTX-6), identification was based on a molecular mass database. An extensive optimization process was carried out to define mass spectrometry parameters and chromatographic conditions for the targeted brevetoxin metabolites. This included the use of reference fragmentation information reported in the literature, particularly for metabolites without commercially available standards but with known molecular masses [5].

To maximize the likelihood of detecting these compounds in the absence of standards, the selected MS/MS transitions were intentionally non-specific (e.g., neutral loss of ammonium adduct and water loss). While this approach allows the screening of a broad range of brevetoxin metabolites, the formal identification of compounds without reference standards cannot be unambiguously confirmed.

### 2.2. Application of Brevetoxin-Specific LC-MS/MS Analysis to Shellfish Samples

The brevetoxin-specific LC-MS/MS analytical method was applied to shellfish samples collected in Corsica (n = 66), as well as additional samples from other locations (n = 20), between January 2018 and December 2023. Analyses were conducted using digestive gland (DG) homogenates from various shellfish species (mussels, oysters, and clams) stored at –80 °C.

Brevetoxin concentrations (C) in the total flesh (TF) were calculated from DG measurements using the actual percentage of DG relative to TF (M_DG_/M_TF_ × 100), according to the following equation: C_TF_ = C_DG_ × (% DG). This extrapolation assumes that brevetoxins are predominantly concentrated in the DG, a hypothesis supported by additional analyses conducted on the remaining flesh (excluding the DG) of contaminated shellfish (mussels and oysters). These analyses confirmed the absence of detectable BTXs non-DG tissues. In fact, a DG-focused analysis enhances the detection of lipophilic toxins at low concentrations, as digestive glands concentrate more of these compounds present in trace amounts.

**Figure 9:**
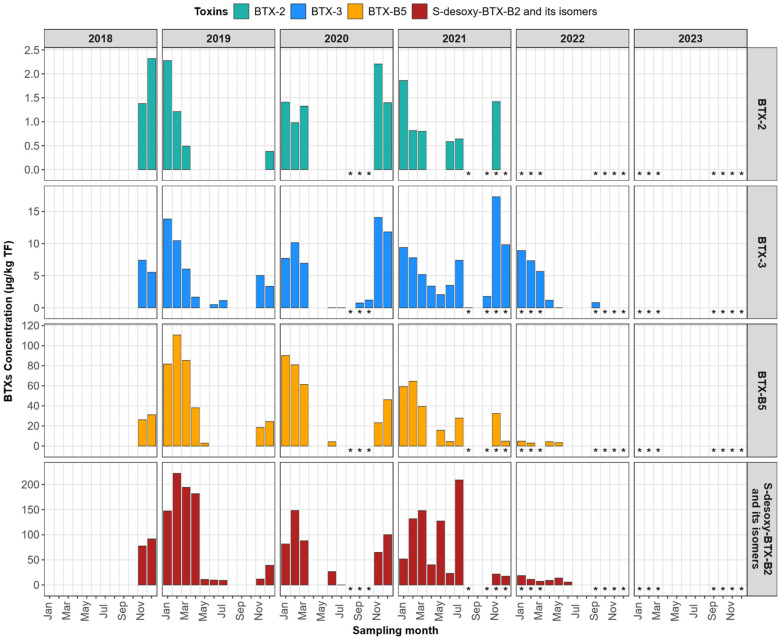
Concentrations of brevetoxin metabolites determined by LC-MS/MS in Corsican shellfish (mussels, * oysters) sampled between January 2018 and December 2023.

Target metabolites were screened using the following approaches:

(i) Commercially available standards: BTX-1, BTX-2; BTX-3, BTX-B5, and S-deoxy-BTX-B2 and its isomer;

(ii) A molecular mass database for metabolites lacking standards with known molecular masses [5]: BTX-B3a, BTX-B3b, BTX-B1, BTX-B2, BTX-B4a, BTX-B4b, and BTX-6. Of all the targeted metabolites by LC-MS/MS, only four brevetoxin metabolites were quantified by LC-MS/MS: BTX-2, BTX-3, BTX-B5, and S-deoxy BTX B2 (including its isomers). Among these, S-deoxy-BTX-B2 and its isomers were predominant, while BTX-2 was present at lower concentrations (Table S4, Figure 9). Contaminated shellfish were primarily harvested during autumn and winter, and all Corsican samples exhibited a consistent qualitative toxin profile.

The brevetoxin concentration was determined by quantifying each detected metabolite (BTX-2, BTX-3, BTX-B5, and S-deoxy-BTX-B2) using their respective certified standards. The concentrations of each analog were then summed to obtain the total brevetoxin concentration, expressed as µg/kg of total flesh (TF). The maximum total brevetoxin concentration was observed in Corsican shellfish samples collected during the autumn/winter period of 2018–2019, with a peak concentration of 345 µg BTX-3 equation/kg TF recorded in February 2019.

Notably, total brevetoxin concentrations in shellfish sampled during January–April 2019, January–February 2020, and February–March and July 2021 exceeded the ANSES guideline value of 180 µg BTX-3 equation/kg TF [33].

### 2.3. Comparison of LC-MS/MS and ELISA Analyses for Brevetoxin Quantification

In parallel with LC-MS/MS analyses, the same shellfish extracts were screened using an enzyme-linked immunosorbent assay (ELISA) (Eurofins Abraxis). Figure S5 provides a detailed description of the assay, including its protocol, characteristics, performance, and limitations.

**Figure 10:**
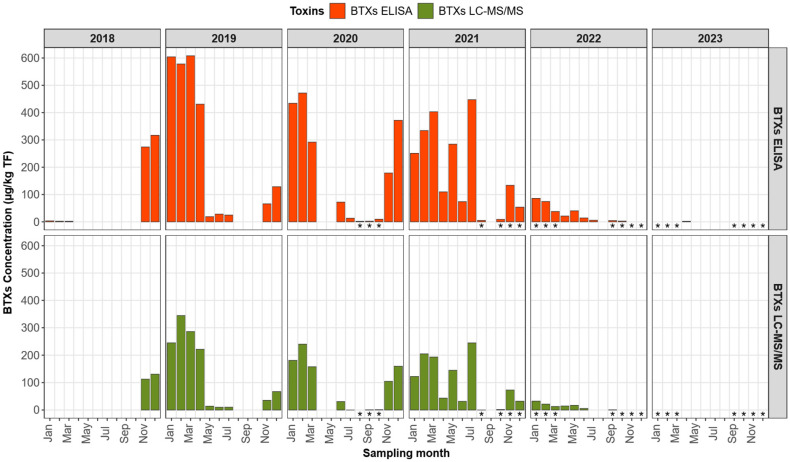
Total brevetoxin concentrations determined using the two approaches (LC MS/MS, ELISA test) in Corsican shellfish (mussels, * oysters) sampled between January 2018 and December 2023.

The ELISA results confirmed the presence of brevetoxins in the same samples where BTX metabolites were quantified by LC-MS/MS (Table S4). Figure 10 compares total brevetoxin concentrations, determined by both methods (LC-MS/MS and ELISA), in Corsican shellfish collected between January 2018 and December 2023. Consistent with the LC-MS/MS results, the ELISA also identified samples with brevetoxin concentrations exceeding the ANSES guideline value of 180 µg BTX-3 equation/kg TF [33], with a maximum concentration of 608 µg BTX-3 equation/kg TF observed in March 2019.

Additionally, brevetoxins were detected in some Corsican samples exclusively by ELISA, at low concentrations (up to 5.8 µg BTX-3 equation/kg TF in July 2022), during the following periods: January–March 2018, July and August 2020, July and October 2022, and April 2023 (Table S4, Figure 10). This discrepancy between the two analytical methods can be attributed to the higher sensitivity of the ELISA. As shown in Figure 10, the concentrations determined by ELISA were systematically higher than those obtained by LC-MS/MS.

**Figure 11:**
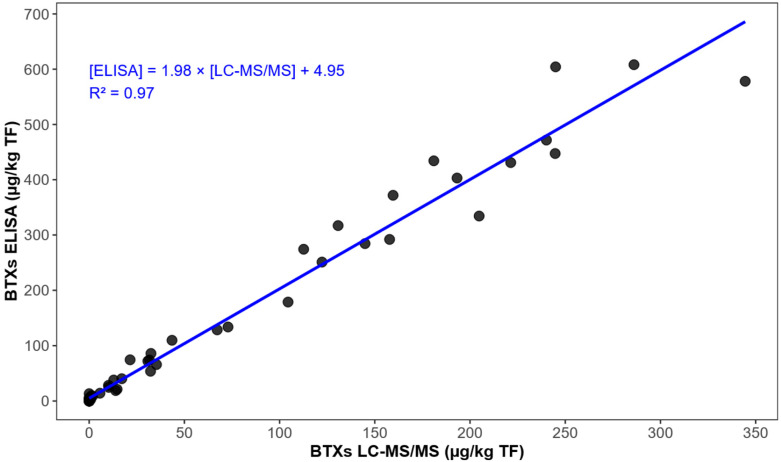
Linear regression comparing brevetoxin concentrations measured by ELISA and targeted LC-MS/MS for paired shellfish samples (r^2^ = 0.97). Each dot represents an individual shellfish sample for which brevetoxin concentrations were measured by both targeted LC-MS/MS (x-axis) and ELISA (y-axis).

In addition to Figure 10, a regression analysis comparing ELISA concentrations to BTX concentrations quantified by LC-MS/MS for paired samples was performed (Figure 11). The methods show a strong correlation (r^2^ = 0.97), with ELISA exhibiting a positive bias compared to LC-MS/MS, as shown by the regression slope of 1.98 and the y-intercept of 4.95.

This representation allows for a clearer interpretation of the relationship between the two methods and facilitates the assessment of the proportion of the ELISA response explained by the BTX targets quantified by LC-MS/MS, as well as the unexplained residual signal, which could be related to other metabolites or cross-reactions. Indeed, this overestimation could be explained by cross-reactivity of the ELISA with certain brevetoxin analogs, particularly S-deoxy-BTX-B2 and its isomer, which exhibit a cross-reactivity of 133%, as well as BTX-B5, with a cross-reactivity of 127%. Furthermore, the potential presence of additional brevetoxin metabolites, not identified by the targeted LC-MS/MS analysis, could also contribute to this discrepancy. On the other hand, an underestimation of known brevetoxins by LC-MS/MS due to poor recovery can be ruled out thanks to the results of fortification experiments on a blank matrix, previously conducted during the laboratory validation of the multi-toxins LC-MS/MS method, in accordance with standard NF V03-110, by establishing an accuracy profile. The average recovery rate of BTX3 ranged from 100.4% to 104.1% depending on the concentration tested, and intralaboratory reproducibility was less than 5% [32]. However, it is possible that some BTX metabolites do not have the same recovery rate as BTX-3. Indeed, it should be noted that only BTX3 could be validated, as BTX2 transformed too rapidly upon contact with the shellfish matrix.

### 2.4. Screening of Shellfish from Non-Corsican Sites by LC-MS/MS and ELISA

Various shellfish species (mussels, oysters, and clams) collected from non-Corsican sites along the French coastline within the EMERGTOX network (20 samples) were analyzed using both LC-MS/MS and ELISA approaches. BTX-3 was the only brevetoxin detected, and it was found at trace levels (below the limit of quantification, BTX-3 < LOQ) in a single sample collected at Banc d’Arguin (Arcachon) in August 2018 (Table S5). This LC-MS/MS result was corroborated by the ELISA test, which indicated a concentration of 5 µg BTX-3 equation/kg TF.

## 3. Discussion

### 3.1. LC-MS/MS and ELISA Approaches for the Detection of Brevetoxin Metabolites for Which Some Standards Are Commercially Available

The coupling of liquid chromatography with tandem mass spectrometry (LC-MS/MS) using an electrospray ionization (ESI) source is the most commonly used analytical method for brevetoxin analysis, for which standards are commercially available [32,35,36,37,38]. However, due to the high metabolism of algal brevetoxins in shellfish, which leads to the formation of numerous metabolites, it was essential to develop a multi-target LC-MS/MS analytical method capable of detecting several brevetoxin metabolites, including those for which no standards are available. We optimized multiple parameters for both mass spectrometry (DP, CE, CXP, transitions, IS, TEM, GS2, GS1, CUR) and chromatography (choice of eluent, column, gradient, temperature, and flow rate). The optimized method relies on the use of the Kinetex XB-C18 column (50 × 2.1 mm, 1.7 µm) and a mobile phase composed of an aqueous eluent (100% H_2_O) and an organic eluent (95% MeOH), each supplemented with 0.2% formic acid and 2 mM ammonium formate. While this method enhances the detection of a wide range of brevetoxin metabolites, the formal identification of metabolites without available standards cannot be guaranteed.

To overcome this limitation, the use of LC/HRMS (high-resolution mass spectrometry), not used directly in this study, can provide detailed structural information on brevetoxin metabolites for which no standards are available. Indeed, previous studies on brevetoxin metabolism have identified numerous new metabolites through rigorous analysis of their fragmentation profiles. However, confirming these structures using advanced tools, such as nuclear magnetic resonance (NMR), requires large quantities of purified samples, which remain challenging to obtain.

In contrast, the ELISA used for brevetoxin quantification offers higher sensitivity than the targeted LC-MS/MS method. Although it does not identify individual metabolites, ELISA detects a broader range of compounds using antibodies targeting the main structure of brevetoxin type B [39], including metabolites not identifiable by the LC-MS/MS. According to the scientific literature, BTX-1 has not been found in shellfish using LC-MS/MS. Given the differences in detection principles and metabolite polarity, high concordance between LC-MS/MS and ELISA results was not expected. Currently, no analytical method has been specifically developed for the detection of hydrophilic brevetoxin forms, suggesting that many weakly lipophilic or hydrophilic analogs may remain unidentified. Moreover, more lipophilic forms than those already characterized cannot be ruled out.

Variability in antibody cross-reactions with brevetoxin congeners likely explain discrepancies between LC-MS/MS and ELISA results. Cross-reactions relative to BTX-3 were as follows: S-deoxy-BTX-B2: 133%; BTX-B5: 127%; BTX-2: 102%; BTX-3: 100%; BTX-9: 83%; and BTX-1: 5%. The minimal cross-reactivity with BTX-1 (and likely its derivatives) poses no significant risk of underestimation in monitoring programs, as BTX-1-like toxins are absent in shellfish. Instead, BTX-2-like toxins dominate the toxic profile of shellfish, accounting for 75% or more of the total brevetoxins present [40]. The rapidity and cost-effectiveness of the ELISA make it an ideal tool for high-throughput screening, as demonstrated in studies on brevetoxin accumulation and depuration dynamics in bivalves [25,40,41,42]. Furthermore, ELISA has been widely employed for monitoring brevetoxin contamination in Florida marine ecosystems [11,39]. However, the ELISA provides only a total concentration of the most abundant brevetoxins in shellfish, without information on metabolite identity or toxicity. Importantly, no cross-reactivity was observed with other shellfish toxins, such as saxitoxins and domoic acid groups (others algal neurotoxins). Additionally, no matrix effects occurred in uncontaminated samples.

### 3.2. Contamination of Shellfish by Brevetoxin Metabolites

Since the establishment of the EMERGTOX network in 2018, only BTX-2 and BTX-3 were detected, because they were the only BTXs monitored, using a broad-spectrum multitoxin LC-MS/MS method, which enables the detection of several groups of regulated and emerging lipophilic toxins in Europe (e.g., okadaic acid, yessotoxins, azaspiracids, brevetoxins, pinnatoxins, etc.) [35]. Following the first detection of BTX-2 and BTX-3 in the French Mediterranean (Corsica) [32], it became necessary to develop a specific LC-MS/MS method for brevetoxin metabolites, selected by ANSES based on toxicity data (see Section 2.2.) [33]. The application of this LC-MS/MS method to all shellfish samples collected in Corsica between January 2018 and December 2023 enabled the quantification of four major metabolites: BTX-2, BTX-3, BTX-B5, and S-deoxy BTX-B2 and its isomers. Among these metabolites, S-deoxy-BTX-B2 and its isomers were predominant, while BTX-2 was present in the minority. This predominance of S-deoxy-BTX-B2 has also been reported in the literature, particularly in Florida, where it was observed in oysters (*Crassostrea virginica*) [40,43] and in clams (*Mercenaria* spp.) [44]. All contaminated French samples exhibited the same toxic profile, with some exceeding the guideline value recommended by ANSES [33], with a maximum of 345 µg BTX-3 equation/kg of total flesh in February 2019 (see Results section).

ELISA testing on these samples also yielded positive results, although with a notable overestimation compared to LC-MS/MS, reaching a maximum concentration of 608 µg BTX-3 equation/kg of total flesh in March 2019. Part of this discrepancy may be attributed to the overestimation of certain metabolites quantified primarily by LC-MS/MS such as S-deoxy-BTX-B2 and its isomers (133% cross-reactivity) and BTX-B5 (127% cross-reactivity). The remaining discrepancy is likely due to the presence of other brevetoxin metabolites not taken into account by LC-MS/MS, as samples from non-Corsican mollusk species tested negative, thus excluding false positive results.

Unlike BTX-1, BTX-2 has occasionally been identified in various shellfish species [21,24,29]. Both toxins are extensively metabolized, and several metabolic pathways have been proposed [28]. For instance, a controlled exposure study of oysters (*Crassostrea virginica*) to pure BTX-2 revealed that BTX-2 accumulated rapidly and was metabolized into several compounds, including BTX-3, the reduced form of BTX-2 [24].

In the French mollusks analyzed here (mussels and oysters), BTX-3 was found at higher levels than BTX-2, consistent with observations in other regions regularly affected by brevetoxins. For example, BTX-3 predominance has been reported in oysters (*Crassostea gigas*) and cockles (*Austrovenus Stutchburyi*) in New Zealand [28,29], as well as in clams (*Mercenaria* spp.) and oysters (*Crassostrea virginica*) in Florida [1,45].

Regarding monitoring programs, the closure and reopening of shellfish production areas in the United States rely on cell counts of *Karenia* spp. or toxin analysis in shellfish tissue, with a regulatory limit of 20 mouse units per 100 g of tissue, determined by mouse bioassay (MBA) or 0.8 mg BTX-2 equivalents/kg of tissue using ELISA [17]. However, these methods do not identify specific toxin analogs. In Australia, production area closures follow different strategies: (i) MBA or (ii) LC-MS/MS to detect BTX-2, BTX-3, BTX-B5, cysteine–BTX-B (S-deoxy-BTX-B2), cysteine–BTX-B sulfoxide (BTX-B2), and taurine–BTX-B (BTX-B1) [46,47,48]. In New Zealand, brevetoxins are regulated with a maximum permissible level of 0.8 mg BTX-2 equivalents/kg, and LC-MS/MS is used as the reference method [49]. In France, BTX-2 and BTX-3 have been monitored since 2018, and since 2023, brevetoxin screening has been extended to other metabolites using the specific LC-MS/MS method developed in this study, targeting BTX-1, BTX-2, BTX-3, BTX-B1, BTX-B2, S-deoxy-BTX-B2, S-deoxy-BTX-B2 isomers, BTX-B3a, BTX-B3b, BTX-B4a, BTX-B4b, BTX-B5, BTX-B6, and BTX-11. ANSES has established a guideline value of 180 µg BTX-3 equivalent/kg of flesh for managing brevetoxin contamination in French shellfish [33].

It is important to note that brevetoxin metabolites in shellfish exhibit varying lipophilic properties compared to their parent compounds. For example, the binding of an amino acid or peptide to the algal toxin decreases lipophilicity, while the binding of a fatty acid increases it. This variability in polarity poses a major challenge for metabolite extraction. Consequently, different authors have emphasized the relevance of using several metabolites as biomarkers of BTX exposure to monitor shellfish toxicity [20,22,44]. Among these, BTX-3, S-deoxy-BTX-B2, BTX-B2, BTX-B1, and BTX-B5 are particularly relevant due to their relative persistence. Nevertheless, given the differences in toxic profiles among shellfish species, appropriate biomarkers must be carefully selected.

## 4. Materials and Methods

### 4.1. Materials

Materials included methanol (LC-MS grade, Carlo Erba, Val de Reuil, France), MilliQ water (Appareil Direct-8, Millipore, Saint Quentin en Yvelines, France), ammonium formate (grade: for mass spectrometry, VWR Chemicals, Fontenay-sous-Bois, France), formic acid (99–100% LC-MS grade, VWR Chemicals, Fontenay-sous-Bois, France), and BTX standards (BTX-1, BTX-2, BTX-3, BTX-5, and S-deoxy-BTX-B2; Novakits, Nantes, France).

Additional materials included glass and plastic containers (for sample storage) and a portable cooler for transporting samples under controlled temperature conditions.

### 4.2. Shellfish

**Figure 12:**
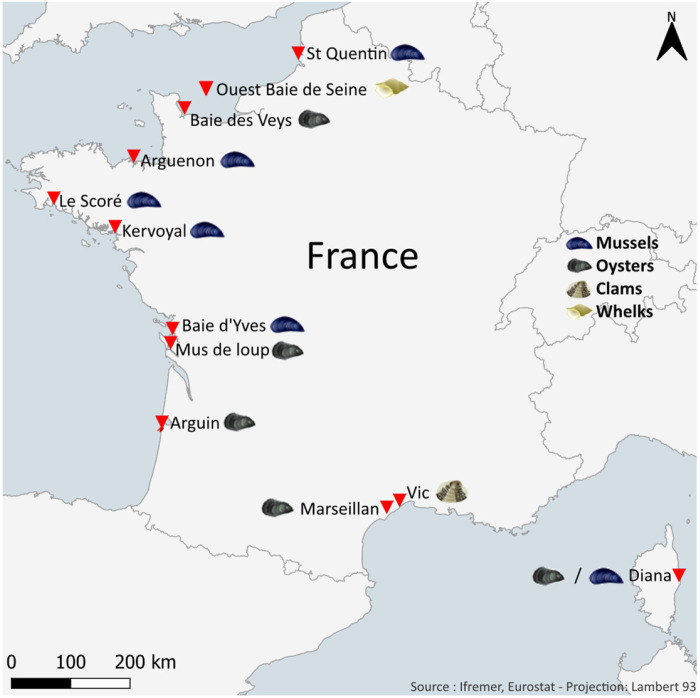
Map of sampling sites in the EMERGTOX network.

Brevetoxins quantification by LC-MS/MS and ELISA tests was performed on threeshellfish taxa: mussels (*Mytilus edulis, Mytilus galloprovincialis*), oysters (*Crassostrea gigas*), and clams (*Ruditapes decussatus*). A total of 2 kg of shellfish was collected monthly from 12 sites along the French coasts, including the English Channel, the Atlantic Ocean, and the Mediterranean Sea (Figure 12).

### 4.3. Methods

#### 4.3.1. Extraction of Brevetoxins (BTXs) from Shellfish

A 200 mg subsample of the homogenized shellfish digestive glands (from the 2 kg pooled sample) was placed in a 2 mL Eppendorf tube containing 250 ± 5 mg of glass beads (100–250 µm diameter). Subsequently, 945 µL of methanol (MeOH) was added. The sample was homogenized using a Mixer Ball Mill (MM400, Retsch GmbH, Haan, Germany) for 2 min at 30 Hz, followed by centrifugation at 15,000 g for 5 min at 4 °C. The supernatant was transferred to a 2 mL volumetric flask, and the extraction process was repeated once more. The combined supernatants were then adjusted to a final volume of 2 mL MeOH. Then, 400 μL of this extract was ultrafiltered (0.2 µm, Nanosep MF, Pall) at 6000 g for 1 min at 4 °C. The filtered extract was then directly analyzed by LC-MS/MS [32].

#### 4.3.2. LC-MS/MS Analysis of BTXs

The BTX-specific LC-MS/MS method used here results from the optimization work detailed earlier in this manuscript (see Section 2 and Section 3). LC-MS/MS analyses were performed on a LC system (UFLC XR, Shimadzu, Marne La Vallee, France) coupled to a hybrid triple quadrupole/linear ion-trap mass spectrometer (API 4000 Qtrap, AB Sciex, Villebon sur Yvette, France) equipped with a heated electrospray ionization (ESI) source.

Brevetoxins were separated using a Kinetex XB-C_18_ (50 × 2.1 mm, 2.6 µm, Phenomenex) with a corresponding core–shell guard column (2.1 mm, Phenomenex) maintained at 30 °C at a flow rate of 0.3 mL/min. The mobile phases consisted of water (A) and methanol/water (95:5, *v*/*v*) (B), each containing 2 mM ammonium formate and 0.2% formic acid. The gradient used was as follows: 30–70% B over 1 min; 70–95% B over 9 min; 95–100% B over 0.1 min; held at 100% B for 1.9 min; returned to initial conditions over 0.1 min, followed by 3.9 min re-equilibration. The injection volume was 5 µL.

MS/MS detection was performed in positive ion mode using multiple reaction monitoring (MRM). Source parameters were as follows: curtain gas 20 psi, ion spray voltage 4500 V, temperature 300 °C, and gases 1 and 2 at 30 psi. The MRM transitions and compound-specific parameters are summarized in Table 2. Limits of detection (LODs) and quantification (LOQs) are indicated in Table S3.

Ionization recoveries and the matrix effects were evaluated by spiking a clean shellfish matrix extract with BTX standards. In brief, 200 mg of uncontaminated shellfish tissue were extracted following the procedure described above. The crude extract was filtered, and 30 µL of mixed standard solution was added to 270 µL of filtered extract. This spiked extract was injected twice (at the start and end of the batch). Field sample concentrations were corrected for ionization recovery accordingly.

#### 4.3.3. ELISA Test for Brevetoxins

From the homogenate of 2 kg of harvested shellfish, a subsample of 1 g of digestive glands was placed in a 40 mL glass vial; subsequently, 9 mL of methanol/MilliQ water (9:1 *v*/*v*) were added. The sample was vigorously shaken by hand for 2 min and then centrifuged for 10 min at 3000 g. The supernatant was transferred to a labeled glass vial. A total of 980 µL of Sample Diluent (provided in the ELISA kit) was pipetted into a labeled 4 mL glass vial, and 20 µL of supernatant were then added (final dilution 1:50 *v*/*v*). The diluted extracts were analyzed as samples by the ELISA test. The brevetoxin concentrations in the samples were determined using external calibration based on a BTX-3 calibration range generated for each analytical batch. Figure S5 summarizes the characteristics, analytical performance, and limitations of the ELISA-BTXs kit used (Novakits, Nantes-France).

## 5. Conclusions

The wide variety of chemical properties of brevetoxin metabolites, combined with the limited number of standards available, complicates the development of robust multi-toxin analytical methods. Nevertheless, many parameters of the brevetoxin-specific analytical method developed here have been optimized and adjusted for both mass spectrometry and liquid chromatography. Its application has enabled the characterization of metabolites present in shellfish since the establishment of the EMERGTOX network in 2018. Of the 14 metabolites screened, 4 were detected and quantified: BTX-2, BTX-3, BTX-B5, and S-deoxy-BTX-B2 and its isomers. The S-deoxy-BTX-B2 form was the predominant metabolite, while BTX-2 was present in very low proportions. All contaminated shellfish samples collected in Corsica displayed the same toxin profile. The ELISA test results show consistently higher brevetoxin concentrations compared to those obtained by targeted LC-MS/MS analysis, reflecting differences in analytical coverage and detection principles between the two methods.

To support risk management and consumer protection, a two-step shellfish monitoring strategy is proposed: (i) initial screening using the ELISA test, which takes into account brevetoxins not quantifiable by LC-MS/MS; and (ii) targeted LC-MS/MS analysis of ELISA-positive samples, which allows confirmatory identification and quantification of specific metabolites. The LCMS method developed here will require full validation by incorporating the missing brevetoxin metabolite standards as soon as they become available. It should be noted that the methods developed so far have mainly focused on brevetoxins produced by *Karenia* sp., and are therefore suitable for the analysis of lipophilic brevetoxins. To achieve a more comprehensive metabolic profile, broader-spectrum extraction protocols will have to be developed, allowing the identification of more hydrophilic metabolites.

## Figures and Tables

**Table 1:** Optimized DP parameters for BTX-1 for [M + H]^+^ and [M + NH_4_]^+^ ions.

Conditions	[M + H]^+^	[M + NH_4_]^+^
1: ACN + 0.1% FA	116	/
2: ACN + 0.2% FA	106	/
3: ACN + 0.3% FA	110	/
4: MeOH + 0.1% FA	110	/
5: MeOH + 0.2% FA	110	/
6: MeOH + 0.3% FA	110	/
7: ACN + 0.2% AF + 2 mM AF	168	88
8: ACN + 0.2% AF + 5 mM AF	170	90
9: MeOH + 0.2% AF + 2 mM AF	166	90
10: MeOH + 0.2% AF + 5 mM AF	168	90
11: MeOH-Isopropanol + 0.2% FA + 2 mM AF	166	90
12: ACN + 6.4 mM NH_4_OH	170	88
13: MeOH + 6.4 mM NH_4_OH	170	90

MeOH: methanol; ACN: acetonitrile; FA: formic acid; AF: ammonium formate; NH_4_OH: ammonium hydroxide.

**Table 2:** Optimized mass spectrometer parameters for targeted toxins.

Toxins	Molecular Formula	Ion Adduct	Q1	DP	Quantification Transition			Qualification Transition		
					Q3	CE	CXP	Q3	CE	CXP
BTX-1 or 11	C_49_H_70_O_13_	[M + NH_4_]^+^	884.5	90	867.5	20	22	849.5	27	22
BTX-2	C_50_H_70_O_14_	[M + NH_4_]^+^	912.5	90	895.5	20	22	877.5	29	24
BTX-3	C_50_H_72_O_14_	[M + NH_4_]^+^	914.5	90	725.5	33	18	825.5	21	20
BTX-B5 or 6	C_50_H_70_O_15_	[M + NH_4_]^+^	928.5	90	911.5	20	22	875.5	31	24
S-deoxy-BTX-B2 or BTX-B1	C_53_H_79_O_16_NS C_52_H_75_O_17_NS	[M + H]^+^	1018.5	180	1000.5	45	24	929.5	47	24
BTX-B2	C_53_H_79_O_17_NS	[M + H]^+^	1034.5	180	1016.5	45	24	929.5	47	24
BTX-B3a	C_64_H_96_O_17_	[M + NH_4_]^+^	1154.7	90	1137.7	20	22	1119.7	28	22
BTX-B3b	C_66_H_100_O_17_	[M + NH_4_]^+^	1182.7	90	1165.7	20	22	1147.7	28	22
BTX-B4a	C_67_H_105_O_18_NS	[M + H]^+^	1244.7	180	1226.7	45	24	929.5	47	24
BTX-B4b	C_69_H_109_O_18_NS	[M + H]^+^	1272.7	180	1254.7	45	24	929.5	47	24

**Table 3:** Optimized source parameters.

Ionization Mode	IS	TEM	GS2	GS1	CUR
Positive	4500	300	30	30	20

## Data Availability

The original results presented in this study are included in the supplementary materials (Table S5). For any questions, please contact the corresponding author.

## Supplementary Materials

The following supporting information can be downloaded at https://www.mdpi.com/article/10.3390/md24020067/s1, Figure S1: MS/MS fragmentation spectra for other brevetoxins with available reference materials (BTX-2, BTX-3, BTX-B5, and S-desoxy-BTX-B2). Figure S2: Evolution of the signal area as a function of the different source parameters: IS (V), TEM (°C)/GS2 (psi), GS2 (psi)/GS1 (psi) for BTX-1 and BTX-2; Table S1: Characteristics of the chromatographic columns tested; Table S2: Concentration of solutions used for linearity verification; Figure S3. Chromatographic profiles of a brevetoxin standard mixture (100 ng/mL) obtained using ten distinct chromatographic columns. Figure S4: Linearity of toxins standards (BTX-1, BTX-2, BTX-3, BTX-5, S-deoxy-BTX-B2) for the five concentration ranges; Table S3: Estimated detection and quantification limits; Table S4: Results of screening, by the two analytical approaches (LC-MS/MS and ELISA test), of samples of Corsican shellfish (mussels, oysters) collected within the framework of the EMERGTOX network during the period January 2018—December 2023; Table S5: Results of screening by the two approaches (LC-MS/MS and ELISA test) of shellfish samples from other sites in the EMERGTOX network, other than the Corsican site; Figure S5: Description, characteristics, performance, and limitations of the ELISA BTXs kit used (Eurofins Abraxis Novakits, Nantes France).
