# Supplementary material for: Brevetoxin Metabolites: Emerging Toxins in French Shellfish Determined by LC-MS/MS and ELISA
Source: Mar Drugs. 2026 Feb 3;24(2):67. doi: 10.3390/md24020067 (PMC12941574; doi:10.3390/md24020067)
Supplement: Supplementary file 1 [file marinedrugs-24-00067-s001.zip › marinedrugs-4048496-supplementary-final version.pdf]

Supplementary material

BTX-2

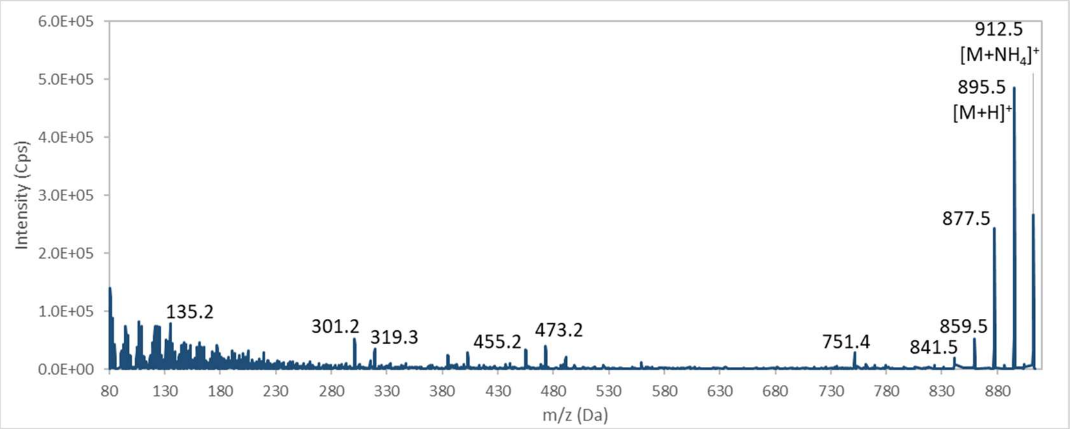

BTX-3

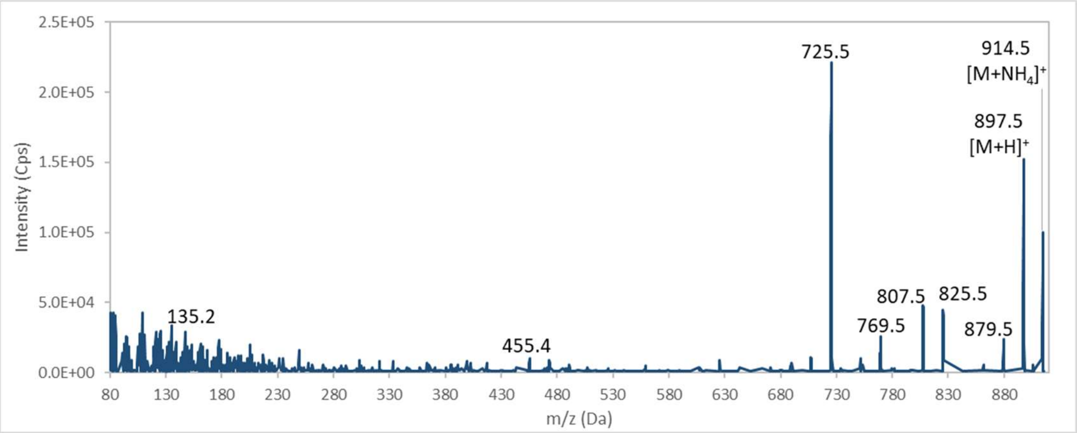

BTX-B5

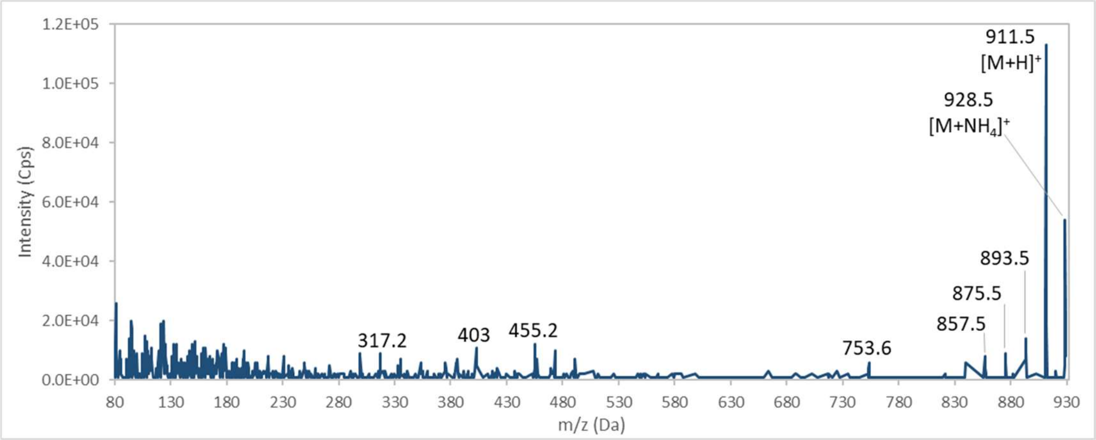

S-desoxy-BTX-B2

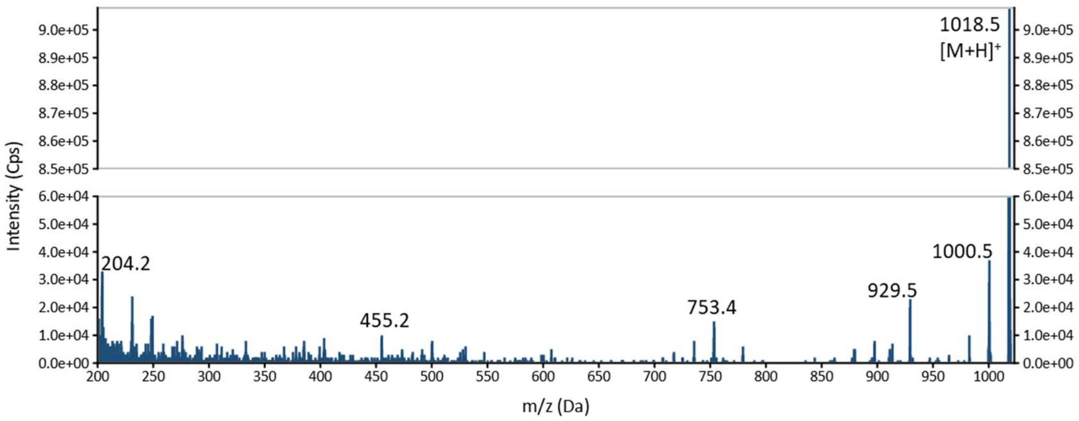

Figure S1. MS/MS fragmentation spectra for other brevetoxins with available reference materials (BTX-2, BTX-3, BTX-B5, and S-desoxy-BTX-B2).

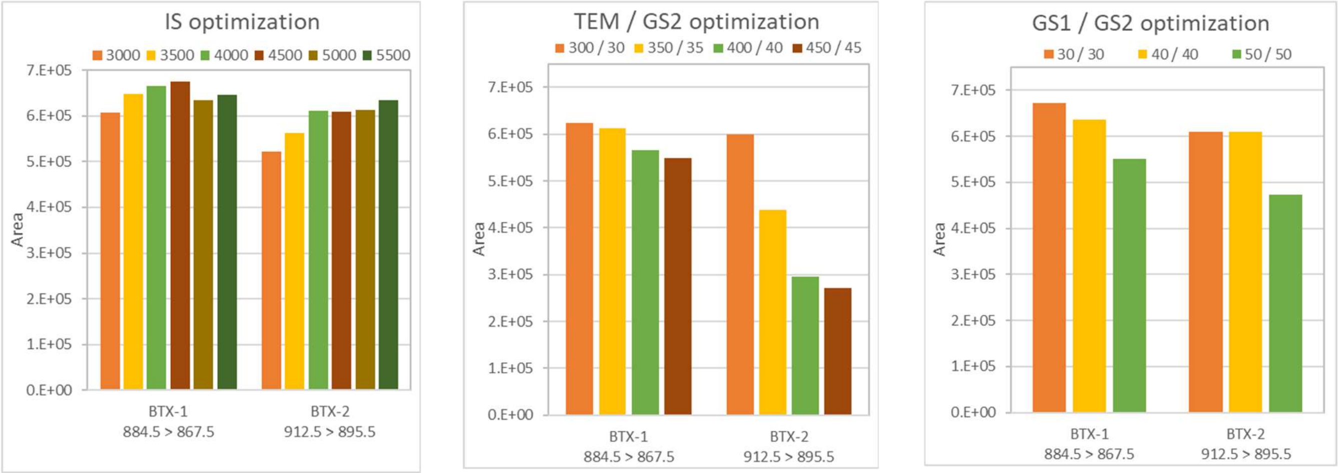

Figure S2. Evolution of the signal area as a function of the different source parameters: IS (V), TEM (°C) / GS2 (psi), GS2 (psi) / GS1 (psi) for BTX-1 and BTX-2.

Table S1: Characteristics of the chromatographic columns tested.

Marque	Phase	Length × Diameter	Particle Size	Pore Size	Carbon Load (%)	Bonding Chemistry
Waters	Acquity BEH C18	100 x 2.1 mm	1.7 µm	130 Å	17%	C18 chain
Phenomenex	Kinetex F5	100 x 2.1 mm	2.6 µm	100 Å	9%	Pentafluorophenylpropyl + trimethylsilane (TMS) endcapping
Phenomenex	Kinetex F5	100 x 2.1 mm	1.7 µm	100 Å	9%	Pentafluorophenylpropyl + trimethylsilane (TMS) endcapping
Phenomenex	Kinetex F5	50 x 2.1 mm	1.7 µm	100 Å	9%	Pentafluorophenylpropyl + trimethylsilane (TMS) endcapping
Phenomenex	Polar C18	50 x 2.1 mm	2.6 µm	100 Å	9%	C18 chain + polar endcapping + trimethylsilane (TMS)
Phenomenex	Kinetex XB-C18	100 x 2.1 mm	2.6 µm	100 Å	10%	C18 chain with isobutyl side chain
Phenomenex	Kinetex XB-C18	100 x 2.1 mm	1.7 µm	100 Å	10%	C18 chain with isobutyl side chain
**Phenomenex**	Kinetex XB-C18	50 x 2.1 mm	1.7 µm	100 Å	10%	C18 chain with isobutyl side chain
Phenomenex	Kinetex C18	100 x 2.1 mm	2.6 µm	100 Å	12%	C18 chain + trimethylsilane (TMS) endcapping
Phenomenex	Kinetex EVO C18	100 x 2.1 mm	2.6 µm	100 Å	11%	C18 chain + trimethylsilane (TMS) endcapping

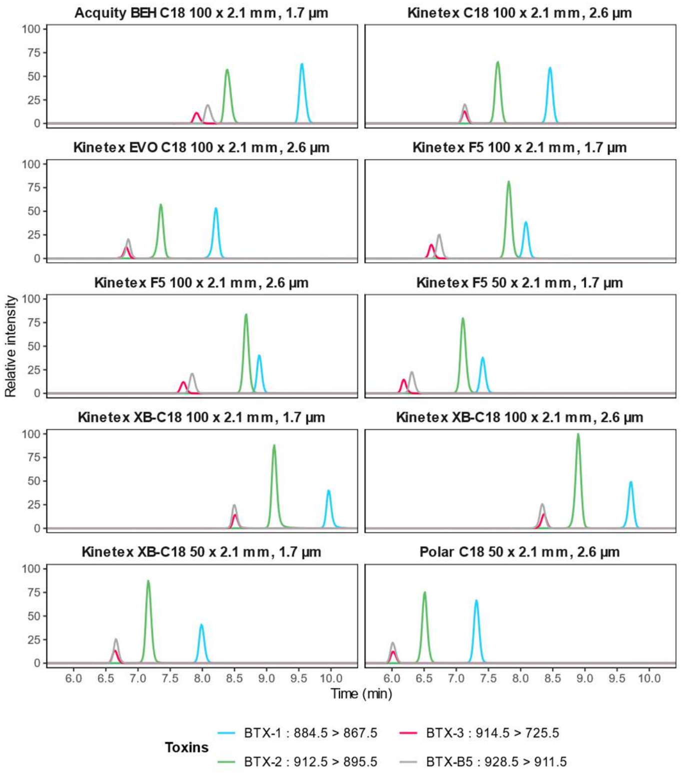

Figure S3. Chromatographic profiles of a brevetoxin standard mixture (100 ng/mL) obtained using ten distinct chromatographic columns.

Table S2: Concentration of solutions used for linearity verification

C° (ng/ml)	C0	C1	C2	C3	C4	C5	C6
BTX-1	0.0	1.00	2.50	7.00	12.50	18.50	25.00
BTX-2	0.0	0.53	1.31	3.68	6.56	9.71	13.13
BTX-3	0.0	1.00	2.50	7.00	12.50	18.50	25.00
BTX-B5	0.0	2.00	5.00	14.00	25.00	37.00	50.00
S-desoxy-BTX-B2	0.0	10.00	20.00	30.00	40.00	50.00	60.00

BTX-1

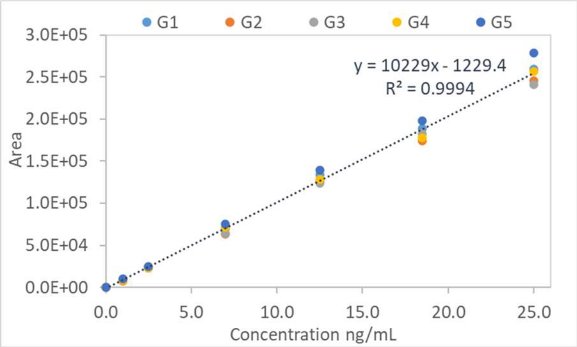

Linearity of BTX-1 for the five concentration ranges

Fisher Test	F_{obs}	$F_{théo}$ (1%)
F_L	3752	7.823
F_{em}	0.67	4.218
$F_L > F_{théo}$: There is a relationship between concentration and areas.<br><br>$F_{em} < F_{théo}$: the chosen model is linear		

Fisher test results for BTX-1

BTX-2

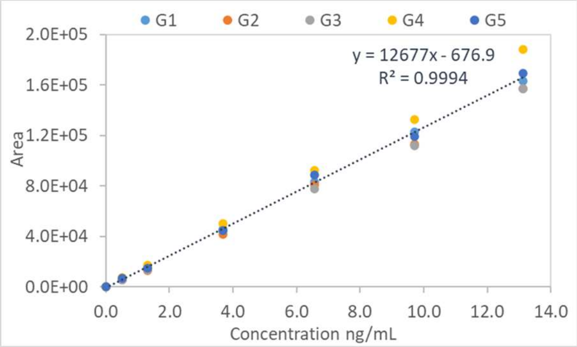

Linearity of BTX-2 for the five concentration ranges

Fisher Test	F_{obs}	$F_{théo}$ (1%)
F_L	2045	7.823
F_{em}	0.37	4.218
$F_L > F_{thé}$: There is a relationship between concentration and areas.<br><br>$F_{em} < F_{théo}$: the chosen model is linear		

Fisher test results for BTX-2

BTX-3

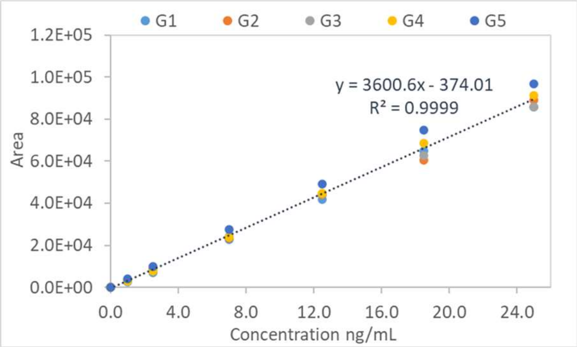

Linearity of BTX-3 for the five concentration ranges

Test Fisher	F_{obs}	$F_{théo}$ (1%)
F_L	2700	7.823
F_{em}	0.03	4.218
$F_L > F_{théo}$: There is a relationship between concentration and areas.<br><br>$F_{em} < F_{théo}$: the chosen model is linear		

Fisher test results for BTX-3

BTX-B5

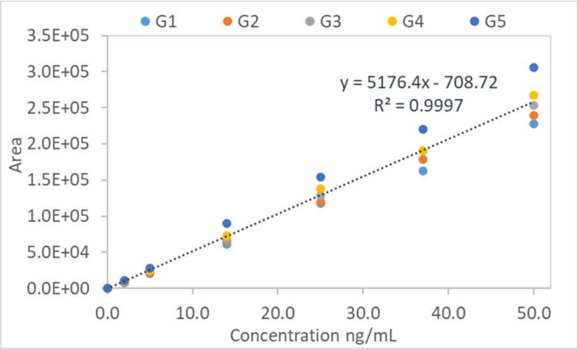

Linearity of BTX-B5 for the five concentration ranges

Test Fisher	F_{obs}	$F_{théo}$ (1%)
F_L	842	7.823
F_{em}	0.07	4.218
$F_L > F_{théo}$: There is a relationship between concentration and areas. $F_{em} < F_{théo}$: the chosen model is linear		

Fisher test results for BTX-B5

S-deoxy-BTX-B2

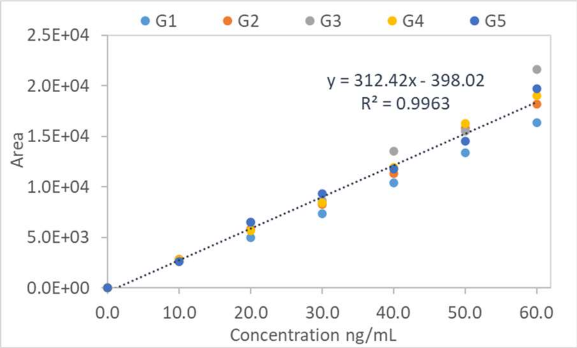

Linearity of S-deoxy-BTX-B2 for the five concentration ranges

Test Fisher	F_{obs}	$F_{théo}$ (1%)
F_L	698	7.823
F_{em}	0.86	4.218
$F_L > F_{théo}$: There is a relationship between concentration and areas. $F_{em} < F_{théo}$: the chosen model is linear		

Fisher test results for S-deoxy-BTX-B2

Figure S4. Linearity of toxins standards (BTX-1, BTX-2, BTX-3, BTX-5, S-deoxy-BTX-B2) for the five concentration ranges.

Table S3: Estimated detection and quantification limits

Toxins	LOD (S/N = 3)		LOQ (S/N = 10)	
	C° ng/ml	C° µg/kg	C° ng/ml	C° µg/kg
BTX-1	0.1	1.0	0.3	3.0
BTX-2	0.07	0.7	0.2	2.0
BTX-3	0.1	1.0	0.3	3.0
BTX-B5	0.3	3.0	1.0	10.0
S-desoxy-BTX-B2	1.0	10.0	3.0	30.0

Table S4. Results of screening, by the two analytical approaches (LC-MS/MS and ELISA test), of samples of Corsican shellfish (mussels, oysters) collected within the framework of the EMERGTOX network during the period January 2018 – December 2023**.**

**Sample references**			**Targeted Brevetoxin (BTX) Metabolites**													**[BTXs] total**	
Code	Matrix	Sampling date (dd/mm/yyyy)	BTX-1	BTX-2	BTX-3	BTX-6	BTX-11	BTX-B1	BTX-B2	S-desoxy-BTX-B2 et isomères	BTX-B3a	BTX-B3b	BTX-B4a	BTX-B4b	BTX-B5	**LC-MS/MS (µg/kg TF)**	**ELISA (µg/kg TF)**
18/004	Mussels	03/01/2018	<LD	<LD	<LD	<LD	***	<LD	<LD	<LD	***	< LD	<LD	<LD	<LD	<LD	**3.40**
18/012	Mussels	05/02/2018	<LD	<LD	<LD	<LD	<LD	<LD	<LD	<LD	<LD	<LD	<LD	<LD	<LD	<LD	**2.30**
18/025	Mussels	05/03/2018	<LD	<LD	<LD	<LD	<LD	<LD	<LD	<LD	<LD	<LD	<LD	<LD	<LD	<LD	**2.20**
18/034	Mussels	03/04/2018	<LD	<LD	<LD	<LD	<LD	<LD	<LD	<LD	<LD	<LD	<LD	<LD	<LD	<LD	<LD
18/046	Mussels	14/05/2018	<LD	<LD	<LD	<LD	<LD	<LD	<LD	<LD	<LD	<LD	<LD	<LD	<LD	<LD	<LD
18/056	Mussels	04/06/2018	<LD	<LD	<LD	<LD	<LD	<LD	<LD	<LD	<LD	<LD	<LD	<LD	<LD	<LD	<LD
18/067	Mussels	02/07/2018	<LD	<LD	<LD	<LD	<LD	<LD	<LD	<LD	<LD	<LD	<LD	<LD	<LD	<LD	<LD
18/111	Mussels	05/11/2018	<LD	7.75	41.64	<LD	<LD	<LD	<LD	436.40	<LD	<LD	<LD	<LD	147.00	**112.60**	**274.20**
18/122	Mussels	10/12/2018	<LD	12.40	29.63	<LD	<LD	<LD	<LD	490.80	<LD	<LD	<LD	<LD	166.20	**130.70**	**317.00**
19/001	Mussels	02/01/2019	<LD	10.35	62.87	<LD	<LD	<LD	<LD	668.80	<LD	<LD	<LD	<LD	371.30	**255.00**	**604.00**
19/012	Mussels	04/02/2019	<LD	6.44	55.62	<LD	<LD	<LD	<LD	1181.60	<LD	<LD	<LD	<LD	588.70	**344.50**	**578.00**
19/023	Mussels	04/03/2019	<LD	2.89	35.72	<LD	<LD	<LD	<LD	1149.50	<LD	<LD	<LD	<LD	504.70	**286.10**	**608.00**
19/034	Mussels	01/04/2019	<LD	<LD	9.87	<LD	<LD	<LD	<LD	1074.60	<LD	<LD	<LD	<LD	225.40	**221.40**	**431.00**
19/049	Mussels	14/05/2019	<LD	<LD	<LD	<LD	<LD	<LD	<LD	49.91	<LD	<LD	<LD	<LD	12.82	**14.00**	**19.00**
19/061	Mussels	17/06/2019	<LD	<LD	3.09	<LD	<LD	<LD	<LD	60.63	<LD	<LD	<LD	<LD	<LD	**10.00**	**28.30**
19/074	Mussels	08/07/2019	<LD	<LD	6.69	<LD	<LD	<LD	<LD	54.04	<LD	<LD	<LD	<LD	<LD	**10.30**	**26.40**
19/108	Mussels	15/10/2019	<LD	<LD	<LD	<LD	<LD	<LD	<LD	<LD	<LD	<LD	<LD	<LD	<LD	<LD	<LD
19/113	Mussels	12/11/2019	<LD	<LD	27.81	<LD	<LD	<LD	<LD	65.28	<LD	<LD	<LD	<LD	102.20	**35.40**	**66.00**
19/125	Mussels	09/12/2019	<LD	2.45	21.60	<LD	<LD	<LD	<LD	252.40	<LD	<LD	<LD	<LD	157.20	**67.20**	**128.60**
20/002	Mussels	06/01/2020	<LD	8.04	44.06	<LD	<LD	<LD	<LD	466.70	<LD	<LD	<LD	<LD	515.40	**181.00**	**434.20**
20/012	Mussels	03/02/2020	<LD	5.88	61.03	<LD	<LD	<LD	<LD	890.30	<LD	<LD	<LD	<LD	486.60	**239.70**	**471.00**
20/023	Mussels	03/03/2020	<LD	6.77	35.44	<LD	<LD	<LD	<LD	449.80	<LD	<LD	<LD	<LD	312.90	**158.00**	**292.00**
20/065	Mussels	08/06/2020	<LD	<LD	<LQ	<LD	<LD	<LD	<LD	172.70	<LD	<LD	<LD	<LD	27.38	**30.80**	**72.20**
20/068	Mussels	06/07/2020	<LD	<LD	<LQ	<LD	<LD	<LD	<LD	<LQ	<LD	<LD	<LD	<LD	<LD	<LD	**13.30**
20/081	Oysters	11/08/2020	<LD	<LD	<LD	<LD	<LD	<LD	<LD	<LD	<LD	<LD	<LD	<LD	<LD	<LD	**2.00**
20/094	Oysters	14/09/2020	<LD	<LD	4.80	<LD	<LD	<LD	<LD	<LD	<LD	<LD	<LD	<LD	<LD	**0.75**	**2.50**

Sample references			Targeted Brevetoxin (BTX) Metabolites													[BTXs] total	
Code	Matrix	Sampling date (dd/mm/yyyy)	BTX-1	BTX-2	BTX-3	BTX-6	BTX-11	BTX-B1	BTX-B2	S-desoxy-BTX-B2 et isomères	BTX-B3a	BTX-B3b	BTX-B4a	BTX-B4b	BTX-B5	**LC-MS/MS (µg/kg TF)**	**ELISA (µg/kg TF)**
20/103	Oysters	12/10/2020	<LD	<LD	5.53	<LD	<LD	<LD	<LD	<LD	<LD	<LD	<LD	<LD	<LD	**1.20**	**9.60**
20/119	Mussels	02/11/2020	<LD	13.30	84.80	<LD	<LD	<LD	<LD	391.70	<LD	<LD	<LD	<LD	139.30	**104.40**	**178.80**
20/127	Mussels	07/12/2020	<LD	9.51	80.44	<LD	<LD	<LD	<LD	681.60	<LD	<LD	<LD	<LD	314.00	**159.60**	**371.80**
21/001	Mussels	04/01/2021	<LD	11.21	56.59	<LD	<LD	<LD	<LD	312.50	<LD	<LD	<LD	<LD	356.30	**122.30**	**251.00**
21/014	Mussels	01/02/2021	<LD	4.38	41.94	<LD	<LD	<LD	<LD	707.80	<LD	<LD	<LD	<LD	346.90	**204.80**	**334.30**
21/027	Mussels	01/03/2021	<LD	5.79	37.57	<LD	<LD	<LD	<LD	1071.40	<LD	<LD	<LD	<LD	285.20	**193.20**	**403.00**
21/037	Mussels	07/04/2021	<LD	<LD	18.18	<LD	<LD	<LD	<LD	215.70	<LD	<LD	<LD	<LD	<LD	**43.50**	**109.70**
21/047	Mussels	03/05/2021	<LD	<LD	12.49	<LD	<LD	<LD	<LD	775.10	<LD	<LD	<LD	<LD	95.71	**145.00**	**284.40**
21/060	Mussels	07/06/2021	<LD	3.48	20.86	<LD	<LD	<LD	<LD	137.30	<LD	<LD	<LD	<LD	26.63	**31.60**	**74.00**
21/072	Mussels	05/07/2021	<LD	4.01	46.25	<LD	<LD	<LD	<LD	1305.50	<LD	<LD	<LD	<LD	173.70	**244.70**	**447.40**
21/087	Oysters	10/08/2021	<LD	<LD	<LQ	<LD	<LD	<LD	<LD	<LD	<LD	<LD	<LD	<LD	<LD	<LD	**5.10**
21/106	Oysters	04/10/2021	<LD	<LD	7.72	<LD	<LD	<LD	<LD	<LD	<LD	<LD	<LD	<LD	<LD	**1.80**	**9.00**
21/119	Oysters	08/11/2021	<LD	5.42	66.09	<LD	<LD	<LD	<LD	83.24	<LD	<LD	<LD	<LD	123.80	**71.60**	**134.00**
21/128	Oysters	06/12/2021	<LD	<LD	40.06	<LD	<LD	<LD	<LD	71.58	<LD	<LD	<LD	<LD	19.92	**32.23**	**53.80**
22/003	Oysters	03/01/2022	<LD	<LD	40.34	<LD	<LD	<LD	<LD	84.51	<LD	<LD	<LD	<LD	21.93	**32.44**	**86.00**
22/017	Oysters	07/02/2022	<LD	<LD	34.60	<LD	<LD	<LD	<LD	52.55	<LD	<LD	<LD	<LD	14.21	**21.50**	**74.50**
22/027	Oysters	07/03/2022	<LD	<LD	30.27	<LD	<LD	<LD	<LD	38.60	<LD	<LD	<LD	<LD	<LD	**12.90**	**37.90**
22/040	Mussels	11/04/2022	<LD	<LD	7.33	<LD	<LD	<LD	<LD	57.61	<LD	<LD	<LD	<LD	26.69	**14.70**	**21.30**
22/051	Mussels	02/05/2022	<LD	<LD	<LQ	<LD	<LD	<LD	<LD	80.93	<LD	<LD	<LD	<LD	19.78	**17.12**	**40.30**
22/060	Mussels	07/06/2022	<LD	<LD	<LD	<LD	<LD	<LD	<LD	35.54	<LD	<LD	<LD	<LD	<LD	**5.70**	**14.10**
22/071	Mussels	04/07/2022	<LD	<LD	<LD	<LD	<LD	<LD	<LD	<LD	<LD	<LD	<LD	<LD	<LD	<LD	**5.80**
22/097	Oysters	05/09/2022	<LD	<LD	3.99	<LD	<LD	<LD	<LD	<LD	<LD	<LD	<LD	<LD	<LD	**0.80**	**4.60**
22/105	Oysters	03/10/2022	<LD	<LD	<LD	<LD	<LD	<LD	<LD	<LD	<LD	<LD	<LD	<LD	<LD	<LD	**2.80**
22/119	Oysters	07/11/2022	<LD	<LD	<LD	<LD	<LD	<LD	<LD	<LD	<LD	<LD	<LD	<LD	<LD	<LD	<LD
22/133	Oysters	05/12/2022	<LD	<LD	<LD	<LD	<LD	<LD	<LD	<LD	<LD	<LD	<LD	<LD	<LD	<LD	<LD
23/001	Oysters	03/01/2023	<LD	<LD	<LD	<LD	<LD	<LD	<LD	<LD	<LD	<LD	<LD	<LD	<LD	<LD	<LD
23/022	Oysters	14/02/2023	<LD	<LD	<LD	<LD	<LD	<LD	<LD	<LD	<LD	<LD	<LD	<LD	<LD	<LD	<LD
23/029	Oysters	14/03/2023	<LD	<LD	<LD	<LD	<LD	<LD	<LD	<LD	<LD	<LD	<LD	<LD	<LD	<LD	<LD
23/045	Mussels	11/04/2023	<LD	<LD	<LD	<LD	<LD	<LD	<LD	<LD	<LD	<LD	<LD	<LD	<LD	<LD	**2.00**

**Sample references**			**Targeted Brevetoxin (BTX) Metabolites**													**[BTXs] total**	
Code	Matrix	Sampling date (dd/mm/yyyy)	BTX-1	BTX-2	BTX-3	BTX-6	BTX-11	BTX-B1	BTX-B2	S-desoxy-BTX-B2 et isomères	BTX-B3a	BTX-B3b	BTX-B4a	BTX-B4b	BTX-B5	**LC-MS/MS (µg/kg TF)**	**ELISA (µg/kg TF)**
23/055	Mussels	11/05/2023	<LD	<LD	<LD	<LD	<LD	<LD	<LD	<LD	<LD	<LD	<LD	<LD	<LD	<LD	<LD
23/065	Mussels	05/06/2023	<LD	<LD	<LD	<LD	<LD	<LD	<LD	<LD	<LD	<LD	<LD	<LD	<LD	<LD	<LD
23/076	Mussels	03/07/2023	<LD	<LD	<LD	<LD	<LD	<LD	<LD	<LD	<LD	<LD	<LD	<LD	<LD	<LD	<LD
23/086	Mussels	31/07/2023	<LD	<LD	<LD	<LD	<LD	<LD	<LD	<LD	<LD	<LD	<LD	<LD	<LD	<LD	<LD
23/101	Oysters	11/09/2023	<LD	<LD	<LD	<LD	<LD	<LD	<LD	<LD	<LD	<LD	<LD	<LD	<LD	<LD	<LD
23/116	Oysters	09/10/2023	<LD	<LD	<LD	<LD	<LD	<LD	<LD	<LD	<LD	<LD	<LD	<LD	<LD	<LD	<LD
23/121	Oysters	06/11/2023	<LD	<LD	<LD	<LD	<LD	<LD	<LD	<LD	<LD	<LD	<LD	<LD	<LD	<LD	<LD
23/133	Oysters	04/12/2023	<LD	<LD	<LD	<LD	<LD	<LD	<LD	<LD	<LD	<LD	<LD	<LD	<LD	<LD	<LD
23/101	Oysters	11/09/2023	<LD	<LD	<LD	<LD	***	<LD	<LD	<LD	***	< LD	<LD	<LD	<LD	<LD	**3.40**
23/116	Oysters	09/10/2023	<LD	<LD	<LD	<LD	<LD	<LD	<LD	<LD	<LD	<LD	<LD	<LD	<LD	<LD	**2.30**
23/121	Oysters	06/11/2023	<LD	<LD	<LD	<LD	<LD	<LD	<LD	<LD	<LD	<LD	<LD	<LD	<LD	<LD	**2.20**
23/133	Oysters	04/12/2023	<LD	<LD	<LD	<LD	<LD	<LD	<LD	<LD	<LD	<LD	<LD	<LD	<LD	<LD	<LD

Table S5. Results of screening by the two approaches (LC-MS/MS and ELISA test) of shellfish samples from other sites in the EMERGTOX network, other than the Corsican site.

**Sample references**				**Targeted Brevetoxin (BTX) Metabolites**													**[BTXs] total**	
Code	Site	Matrix	Sampling date (dd/mm/yyyy)	BTX1	BTX2	BTX3	BTX6	BTX11	BTX-B1	BTX-B2	S-deoxy-BTX-B2 et isomères	BTX-B3a	BTX-B3b	BTX-B4a	BTX-B4b	BTX-B5	**LC-MS/MS (µg/kg CT)**	**ELISA (µg/kg CT)**
18/087	Arguin	Mussels	13/08/2018	<LD	<LD	**< LQ**	<LD	<LD	<LD	<LD	<LD	<LD	<LD	<LD	<LD	<LD	**< LQ**	**5.00**
18/095	Arguin	Mussels	10/09/2018	<LD	<LD	<LD	<LD	<LD	<LD	<LD	<LD	<LD	<LD	<LD	<LD	<LD	<LD	<LD
19/056	Arguin	Mussels	03/06/2019	<LD	<LD	<LD	<LD	<LD	<LD	<LD	<LD	<LD	<LD	<LD	<LD	<LD	<LD	<LD
19/067	Arguin	Mussels	01/07/2019	<LD	<LD	<LD	<LD	<LD	<LD	<LD	<LD	<LD	<LD	<LD	<LD	<LD	<LD	<LD
19/078	Arguin	Mussels	05/08/2019	<LD	<LD	<LD	<LD	<LD	<LD	<LD	<LD	<LD	<LD	<LD	<LD	<LD	<LD	<LD
19/097	Arguin	Mussels	12/09/2019	<LD	<LD	<LD	<LD	<LD	<LD	<LD	<LD	<LD	<LD	<LD	<LD	<LD	<LD	<LD
20/112	Arguenon	Mussels	02/11/2020	<LD	<LD	<LD	<LD	<LD	<LD	<LD	<LD	<LD	<LD	<LD	<LD	<LD	<LD	<LD
18/057	Le Scoré	Mussels	04/06/2018	<LD	<LD	<LD	<LD	<LD	<LD	<LD	<LD	<LD	<LD	<LD	<LD	<LD	<LD	<LD
18/092	Le Scoré	Mussels	10/09/2018	<LD	<LD	<LD	<LD	<LD	<LD	<LD	<LD	<LD	<LD	<LD	<LD	<LD	<LD	<LD
19/051	Le Scoré	Mussels	22/05/2019	<LD	<LD	<LD	<LD	<LD	<LD	<LD	<LD	<LD	<LD	<LD	<LD	<LD	<LD	<LD
19/068	Le Scoré	Mussels	02/07/2019	<LD	<LD	<LD	<LD	<LD	<LD	<LD	<LD	<LD	<LD	<LD	<LD	<LD	<LD	<LD
20/101	Le Scoré	Mussels	07/10/2020	<LD	<LD	<LD	<LD	<LD	<LD	<LD	<LD	<LD	<LD	<LD	<LD	<LD	<LD	<LD
19/076	Marseillan	Oysters	08/07/2019	<LD	<LD	<LD	<LD	<LD	<LD	<LD	<LD	<LD	<LD	<LD	<LD	<LD	<LD	<LD
19/096	Marseillan	Oysters	09/09/2019	<LD	<LD	<LD	<LD	<LD	<LD	<LD	<LD	<LD	<LD	<LD	<LD	<LD	<LD	<LD
19/072	Kervoyal	Mussels	01/07/2019	<LD	<LD	<LD	<LD	<LD	<LD	<LD	<LD	<LD	<LD	<LD	<LD	<LD	<LD	<LD
20/062	Kervoyal	Mussels	22/06/2020	<LD	<LD	<LD	<LD	<LD	<LD	<LD	<LD	<LD	<LD	<LD	<LD	<LD	<LD	<LD
19/062	Mus de loup (a)	Oysters	04/06/2019	<LD	<LD	<LD	<LD	<LD	<LD	<LD	<LD	<LD	<LD	<LD	<LD	<LD	<LD	<LD
19/071	Baie d'Yves (a)	Mussels	02/07/2019	<LD	<LD	<LD	<LD	<LD	<LD	<LD	<LD	<LD	<LD	<LD	<LD	<LD	<LD	<LD
20/014	Mus de loup (a)	Oysters	10/02/2020	<LD	<LD	<LD	<LD	<LD	<LD	<LD	<LD	<LD	<LD	<LD	<LD	<LD	<LD	<LD
20/072	Mus de loup (a)	Oysters	21/07/2020	<LD	<LD	<LD	<LD	<LD	<LD	<LD	<LD	<LD	<LD	<LD	<LD	<LD	<LD	<LD

Importance of Brevetoxin Determination

Neurotoxic shellfish poisoning (NSP) is caused by polyether toxins known as Brevetoxins. Brevetoxins (PbTxs) are produced by the dinoflagellate *Karenia brevis,* which causes harmful algal blooms (HABs) know as red tides. The Brevetoxins are toxic to fish, marine mammals, birds and humans, but not to shellfish. Contamination of shellfish with Brevetoxin has been associated with the presence of harmful algal blooms in various parts of the world.

Mortality events attributed to HABs have been documented for fish, manatee, dolphins, and seabirds. In man, NSP causes dose-dependent symptoms of diarrhea, nausea, and vomiting, chills, sweats, reversal of temperature, hypotension, arrhythmias, numbness, tingling, bronchoconstriction, paralysis, seizures, and coma.

The Brevetoxin ELISA allows the determination of 40 samples in duplicate determination. Only a few milliliters of sample are required. The test can be performed in less than 2 hours.

Performance Data

Test sensitivity: The limit of detection for Brevetoxin is calculated as: Xn +/- 3SD (n=20) and is equal to 0.005 ng/ml in water and 2.25 ng/gm in diluted shellfish (when using a dilution factor of 450). The concentration of residue necessary to cause 50% inhibition (50% B/B_0) is approximately 0.16 ng/mL. Determinations closer to the middle of the calibration range of the test yield the most accurate results.

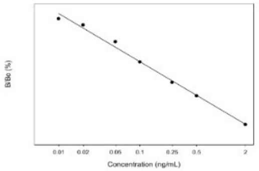

Test reproducibility: Coefficients of variation (CVs) for standards: <10%, CVs for samples: <15%.

Selectivity: This ELISA recognizes Brevetoxin and other NSP toxins to varying degrees:

Cross-reactivities:

PbTx-3	100%
Desoxy PbTx-2	133%
PbTx-5	127%
PbTx-2	102%
PbTx-9	83%
PbTx-6	13%
PbTx-1	5%

No cross-reactivity was shown with any of the following common PSP shellfish toxins: saxitoxin, neosaxitoxin, dc-STX, gonyautoxins-1/4, gonyautoxins-2/3, B-2; B-1; C-1/2 and domoic acid.

Samples: Salt Water and shellfish samples (after recommended dilution) were tested for matrix effects in the ELISA. No matrix effects were determined.

General Limited Warranty: Eurofins Abraxis warrants the products manufactured by the Company, against defects and workmanship when used in accordance with the applicable instructions for a period not to extend beyond the product's printed expiration date. **Eurofins Abraxis makes no other warranty, expressed or implied. There is no warranty of merchantability or fitness for a particular purpose**

Contact:

Novakits
Tests et Standards Contaminants
40 Bd Jean Ingres / 44100 NANTES / France
Tel: 09 61 58 14 40 / Mail: info@novakits.com

Eurofins Abraxis
124 Railroad Drive
Warminster, PA 18974
Tel.: (215) 357-3911
Fax: (215) 357-5232
Email: info.ET.Warminster@eurofinsus.com
WEB: www.abraxiskits.com

R09282020

Brevetoxin (NSP) ELISA, Microtiter Plate

Enzyme-Linked Immunosorbent Assay for the Determination of Brevetoxin (NSP) in Water and Contaminated Samples

Product No. 520026

1. General Description

The Brevetoxin ELISA is an immunoassay for the quantitative and sensitive detection of Brevetoxin. Brevetoxin is one of the toxins associated with neurotoxic shellfish poisoning (NSP). This test is suitable for the quantitative and/or qualitative detection of Brevetoxin in water samples as well as shellfish samples. Sea water samples require addition of a pre-treatment solution and shellfish samples require an extraction (see Preparation of Samples, Section C) prior to analysis. If necessary, positive samples can be confirmed by HPLC, GC/MS, or other conventional methods.

2. Safety Instructions

The standard solutions in this test kit contain small amounts of Brevetoxin (PbTx-3). In addition, the substrate (color) solution contains tetramethylbenzidine and the stop solution contains diluted sulfuric acid. Avoid contact of stopping solution with skin and mucous membranes. If these reagents come in contact with the skin, wash with water.

3. Storage and Stability

The Brevetoxin ELISA should to be stored in the refrigerator (2-8°C). The solutions must be allowed to reach room temperature (20-25°C) before use. Reagents may be used until the last day of the month as indicated by the expiration date on the box.

4. Test Principle

The test is a direct competitive ELISA based on the recognition of Brevetoxin by specific antibodies. Brevetoxin, when present in a sample, and a Brevetoxin enzyme-conjugate compete for the binding sites of sheep anti-Brevetoxin antibodies that have been immobilized in the wells of a microtiter plate. After a washing step and addition of the substrate solution, a color signal is produced. The intensity of the blue color is inversely proportional to the concentration of Brevetoxin present in the sample. The color reaction is stopped after a specified time and the color is evaluated using an ELISA reader. The concentrations of the samples are determined by interpolation using the standard curve constructed with each run.

5. Limitations of the Brevetoxin ELISA, Possible Test Interference

Numerous organic and inorganic compounds commonly found in samples have been tested and found not to interfere with this test. However, due to the high variability of compounds that might be found in samples, test interferences caused by matrix effects can not be completely excluded.

Mistakes in handling the test can also cause errors. Possible sources for such errors can be: Inadequate storage conditions of the test kit, incorrect pipetting sequence or inaccurate volumes of the reagents, too long or too short incubation times during the immune and/or substrate reaction, exposure to direct or indirect sunlight during the substrate reaction, or extreme temperatures during the test performance (lower than 10°C or higher than 30°C).

The Eurofins Abraxis Brevetoxin ELISA kit provides screening results. As with any analytical technique (GC/MS, HPLC, etc.) positive samples requiring some action should be confirmed by an alternative method.

Working Instructions

A. Materials Provided

1. Microtiter plate coated with sheep anti-Brevetoxin
2. Standards PbTx-3 (8): 0, 0.010, 0.025, 0.05, 0.1, 0.25, 0.5, 2.0 ng/mL, 1 mL each
3. Brevetoxin-HRP Conjugate, 6 mL
4. Sample Diluent (1X), 2 X 30 mL, used to dilute samples
5. Wash Buffer (5X) Concentrate, 100 mL, must be diluted before use, see Test Preparation (Section D)
6. Substrate (Color) Solution (TMB), 12 mL
7. Stop Solution, 12 mL
8. Sea Water Pretreatment Solution, 25 mL

B. Additional Materials (not delivered with the test kit)

1. Micro-pipettes with disposable plastic tips (10-200, and 200-1000 µL)
2. Multi-channel pipette (10-250 µL) or stepper pipette with plastic tips (10-250 µL), or electronic repeating pipette with disposable plastic tips (capable of delivering 50-250 µL)
3. Microtiter plate washer (optional)
4. Deionized or distilled water
5. Container with 500 mL capacity (for diluted 1X Wash Buffer, see Test Preparation, Section D)
6. Microtiter plate reader (wave length 450 nm)
7. Paper towels or equivalent absorbent material
8. Timer
9. Materials and reagents for Sample Preparation (see Section C)

C. Preparation of Samples

Shellfish (Mussels, Clams, Oysters)

1. Remove the shellfish from their shells, wash with deionized water, drain and blot dry. Homogenize using a Waring blender, Polytron or equivalent.
2. Weigh a 1.0 g portion of the homogenized shellfish into an appropriately labeled 40 mL glass vial.
3. Add 9.0 mL of methanol/deionized water (9:1 v/v) solution.
4. Cap vial and shake by hand vigorously for 2 minutes.
5. Centrifuge mixture for 10 minutes at 3000 x g. Collect and transfer the supernatant to an appropriately labeled glass vial.
6. Add 980 µL of Sample Diluent to an appropriately labeled 4 mL glass vial. Add 20 µL of the collected extract (from Step 5) to the Sample Diluent (equals a 1:50 dilution).
7. Analyze diluted extracts as samples (Assay Procedure, Section F, Step 1).

The Brevetoxin concentration contained in the samples is determined by multiplying the ELISA result by a factor of 450. Highly contaminated samples outside the range of the curve should be diluted further and re-analyzed. Additional Sample Diluent (PN 205226) can be purchased from Eurofins Abraxis.

Sea Water

1. Collect 2 mL of sea water sample in a 4 mL glass vial.
2. To prevent loss of Brevetoxin to the glass surface, immediately add 0.5 mL of Sea Water Pretreatment Solution, mix by hand. (Alternatively, a larger sea water sample can be collected in a glass container and 0.5 mL of Sea Water Pretreatment Solution added for every 2 mL of sample.)
3. Analyze preserved sample as samples (Assay Procedure step 1)

The Brevetoxin concentration contained in the sea water sample is determined by multiplying the ELISA result by a factor of 1.25. Highly contaminated samples outside the range of the curve should be diluted in Sample Diluent (PN 205226), and re-analyzed. Additional Sample Diluent or Sea Water Pretreatment Solution (PN 205227) can be purchased from Eurofins Abraxis.

D. Test Preparation

Micro-pipetting equipment and pipette tips for pipetting the standards and the samples are necessary. We recommend using a multi-channel pipette or a stepping pipette for adding the conjugate, the substrate solution and the stop solution in order to equalize the incubations periods of the standard solutions and the samples on the entire microtiter plate. Please use only the reagents and standards from one package lot in one test, as they have been adjusted in combination.

1. Adjust the microtiter plate and the reagents to room temperature before use.
2. Remove the number of microtiter plate strips required from the foil bag. The remaining strips are stored in the foil bag and zip-locked closed. Store the remaining kit in the refrigerator (2-8°C).
3. The standards, enzyme conjugate, substrate (color) and stop solutions are ready to use and do not require any further dilutions.
4. Dilute the Wash Buffer (5X) Concentrate) at a ratio of 1:5. If using the entire bottle (100 mL), add to 400 mL of deionized or distilled water.
5. The stop solution should be handled with care as it contains diluted H_2SO_4.

E. Working Scheme

The microtiter plate consists of 12 strips of 8 wells, which can be used individually for the test. The standards must be run with each test. Never use the values of standards which have been determined in a test performed previously.

Std 0-Std 7: Standards
0; 0.010; 0.025; 0.05; 0.1; 0.25, 0.5; 2.0 (ng/mL) or ppb

Sam1, Sam2, etc.: Samples

	1	2	3	4	5	6	7	8	9	10	11	12
A	Std 0	Std 4	Sam 1	etc.								
B	Std 0	Std 4	Sam 1									
C	Std 1	Std 5	Sam 2									
D	Std 1	Std 5	Sam 2									
E	Std 2	Std 6	etc.									
F	Std 2	Std 6	etc.									
G	Std 3	Std 7										
H	Std 3	Std 7										

F. Assay Procedure

1. Add **50 µL of standards, samples (water) or sample extracts (shellfish)** into the wells of the test strips according to the working scheme given. Analysis in duplicate or triplicate is recommended.
2. Add **50 µL of enzyme conjugate solution** to the individual wells successively using a multi-channel, stepping, or electronic repeating pipette. Cover the wells with parafilm or tape and mix the contents by moving the strip holder in a rapid circular motion on the benchtop for about 30 seconds. Be careful not to spill contents. Incubate for **60 minutes**.
3. After incubation, remove the covering, decant the contents of the wells into a sink, and blot the inverted plate on a stack of paper towels. Wash the strips **three times** using the diluted wash buffer. Use at least a volume of **250 µL of 1X wash buffer** for each well and each washing step. ***Blot the inverted plate after each wash step*** on a stack of paper towels. After the last wash/blot, check the wells for any remaining buffer in the wells, and if necessary, remove by additional blotting.
4. Add **100 µL of substrate (color) solution** to the wells successively using a multi-channel, stepping, or electronic repeating pipette. Incubate the strips for **30 minutes** at room temperature. Protect the strips from direct sunlight.
5. Add **100 µL of stop solution** to the wells in the same sequence as for the substrate (color) solution using a multi-channel, stepping, or electronic repeating pipette.
6. Read the absorbance at 450 nm using a microplate ELISA photometer within 15 minutes after the addition of the stopping solution.

G. Evaluation

The evaluation of the ELISA can be performed using commercial ELISA evaluation programs (4-Parameter (preferred) or Logit/Log). For manual evaluation, calculate the mean absorbance value for each of the standards. Calculate the $\%B/B_0$ for each standard by dividing the mean absorbance value for each standard by the Zero Standard (Standard 0) mean absorbance. Construct a standard curve by plotting the $\%B/B_0$ for each standard on the vertical linear (y) axis versus the corresponding Brevetoxin concentration on the horizontal logarithmic (x) axis on graph paper. $\%B/B_0$ for samples will then yield levels in ppb of Brevetoxin by interpolation using the standard curve.

The concentrations of the samples are determined using the standard curve run with each test. Samples showing lower concentrations of Brevetoxin compared to standard 1 (0.01 ng/mL) are considered as negative. Samples showing a higher concentration than standard 7 (2.0 ng/mL) must be diluted further to obtain more accurate results. Results must be multiplied by the appropriate dilution factor (see Sample Preparation, Section C).

As with any analytical technique (GC/MS, HPLC, etc.) samples requiring regulatory action should be confirmed by an alternative method.

Figure S5. description, characteristics, performance, and limitations of the ELISA-BTXs kit used (Eurofins Abraxis - Novakits, Nantes-France).
